# Extraction of Expansion Trees

**DOI:** 10.1007/s10817-018-9453-9

**Published:** 2018-01-27

**Authors:** Alexander Leitsch, Anela Lolic

**Affiliations:** 1 Institute of Computer Languages (E185), Vienna University of Technology, Favoritenstrasse 9, 1040 Vienna, Austria; 2 Institute of Discrete Mathematics and Geometry, Vienna University of Technology, Wiedner Hauptstrasse 8 - 10, 1040 Vienna, Austria

**Keywords:** Cut-elimination, Proof mining, Herbrand sequent, Expansion tree

## Abstract

We define a new method for proof mining by CERES (cut-elimination by resolution) that is concerned with the extraction of expansion trees in first-order logic (see Miller in Stud Log 46(4):347–370, 1987) with equality. In the original CERES method expansion trees can be extracted from proofs in normal form (proofs without quantified cuts) as a post-processing of cut-elimination. More precisely they are extracted from an ACNF, a proof with at most atomic cuts. We define a novel method avoiding proof normalization and show that expansion trees can be extracted from the resolution refutation and the corresponding proof projections. We prove that the new method asymptotically outperforms the standard method (which first computes the ACNF and then extracts an expansion tree). Finally we compare an implementation of the new method with the old one; it turns out that the new method is also more efficient in our experiments.

## Introduction

Proof analysis and proof mining are central mathematical activities. Extracting additional mathematical information from existing proofs plays an important role in the process of proof mining. Mathematical proofs in general are based on the structuring of reasoning by intermediate statements (lemmas). The drawback of the use of lemmas is that only their truth but not their proofs are reflected in the derivation of their end-sequents. These proofs, however, may contain important mathematical information which can be extracted only from the proofs of these lemmas. One of the most important theorems in mathematical logic is Gentzen’s Hauptsatz [15]. It states that lemmas (cuts) can be eliminated from first-order derivations, resulting in a lemma-free proof combining all subproofs of the original derivation.

The result of cut-elimination is a purely combinatorial proof. These combinatorial proofs can be used to extract explicit mathematical information. Proofs can be transformed in a way such that this information becomes visible. Such a transformation for cut-free **LK**-proofs of prenex end-sequents was given by Gentzen [15] in the mid-sequent theorem. It basically states that a proof $\varphi$ can be transformed into a proof $\varphi '$ such that $\varphi '$ contains a so-called mid-sequent, that splits the proof into a propositional part and a part with quantifier inferences. The mid-sequent is propositionally valid and contains the instantiations of the quantifiers needed to prove the end-sequent. These instantiations may contain crucial mathematical information.

Cut-elimination plays a key role in the analysis of mathematical proofs. A prominent example is Girard’s analysis (see in [16]) of Fürstenberg and Weiss’ topological proof [14] of van der Waerden’s theorem [27] on partitions. After cut-elimination was applied to the proof of Fürstenberg and Weiss, the result was van der Waerden’s original elementary proof.

Girard’s analysis of the proof of Fürstenberg and Weiss was carried out by hand within mathematical meta-language. However, in automated proof analysis, formal proofs are vital. Therefore the first step in automated proof analysis consists in formalizing the mathematical proofs (typically expressed in traditional mathematical language). The next steps are algorithmic cut-elimination and, finally, the interpretation of the resulting formal proof.

For automated proof analysis of mathematical proofs, the cut-elimination method CERES (Cut-Elimination by RESolution) was developed (see [4, 5]). CERES substantially differs from the traditional reductive cut-elimination methods a la Gentzen. In the reductive methods cuts are eliminated by stepwise reduction of cut-complexity. These methods always identify the uppermost logical operator in the cut-formula and either eliminate it directly (grade reduction) or indirectly (rank reduction). It is typical for such a method that the cut formulas are “peeled” from the outside till only atomic cuts are left. These methods are local in the sense that only a small part of the whole proof is analyzed, namely the derivation corresponding to the introduction of the uppermost logical operator. As a consequence, many types of redundancy in proofs are left undetected in the reductive methods, leading to an unfortunate computational behavior. In contrast, the method CERES to be presented in Sect. 3 is based on a structural analysis of the whole proof. Here all cut-derivations in an **LK**-proof $\varphi$ of a sequent *S* are analyzed simultaneously. The interplay of binary rules, which produce ancestors of cut formulas and those which do not, defines a structure which can be represented as a set of clauses $\mathrm{CL}(\varphi )$. $\mathrm{CL}(\varphi )$ is always unsatisfiable and thus admits resolution refutations. A resolution refutation $\gamma$ of $\mathrm{CL}(\varphi )$ may serve as a skeleton of an **LK**-proof of *S* with only atomic cuts. The proof itself (a CERES normal form) is obtained by replacing clauses in $\gamma$ by so-called proof projections of $\varphi$. To handle predicate logic with equality the calculi can be extended by equality rules; instead of resolution refutations we obtain refutations by resolution and paramodulation (for details see [2]). CERES is a semi-semantical method of cut-elimination (see [7]). A detailed description of CERES, a comparison with reductive methods, its extensions and a complexity analysis of the method can be found in the book [6].

CERES has been applied to real mathematical proofs. The most interesting application was the analysis of Fürstenberg’s proof of the infinitude of primes [13] where, as a result of cut-elimination by CERES, Euclid’s original argument of prime construction was obtained.

The last step in automated proof analysis consists in the interpretation of the result. In this interpretation it is crucial to obtain compact and meaningful information rather than a full (and typically very long) formal proof. The relevant information can be bounds for variables that are used in the proof or even programs representing its algorithmic content. Actually, it is possible to extract functionals, based on Gödel’s dialectica interpretation [17], and construct programs from proofs in Peano arithmetic; see [8, 9] for applications to mathematical proofs.

Another structure representing explicit information are mid-sequents (also called Herbrand sequents). Herbrand’s theorem, [10, 18], provides one of the most fundamental insights of logic and characterizes the validity of a formula in classical first-order logic by the existence of a propositional tautology composed of instances of that formula. Roughly speaking, Herbrand sequents are compact structures encoding the essence of proofs with prenex end-sequents. Hence in mathematical proof analysis it is frequently more important to extract Herbrand sequents than full formal proofs (which may be too large to be interpreted). There are efficient algorithms for extracting Herbrand sequents from cut-free proofs, see e.g. [20]. Though every formula (and any sequent) can be transformed to prenex form such a transformation is unnatural and can have a disastrous impact on proof complexity (see [3]). Thus it is vital to extend the methods to non-prenex formulas and sequents. Miller [23] developed the structure of expansion trees (and expansion proofs) generalizing the derivation of end-sequents from a mid-sequent in the prenex case. The so-called deep function of an expansion proof generalizes the mid-sequent itself. As expansion proofs abstract from propositional reasoning they provide compact and explicit information about the mathematical content of formal cut-free proofs.

The result of the method CERES is a CERES normal form, which is a (typically very long) formal proof with at most atomic cuts. This proof can then be used for further investigation and particularly for the extraction of Herbrand sequents and expansion proofs in order to obtain compact information. In the ordinary CERES-method, expansion proofs can be extracted from an ACNF. In this paper we show that even the construction of an ACNF can be avoided in computing the expansion proofs. In particular, we prove that the expansion proof of the CERES normal form can be constructed from the partial expansion proofs of the projections obtained by CERES, after deleting the clause parts. A ground refutation of the characteristic clause set and the projections suffice for the extraction of expansion proofs making the construction of the CERES normal form itself obsolete. This improvement yields a gain in asymptotic complexity. In particular we show that the new method outperforms the old one (quadratic versus cubic) and that the complexity of the new method can never be higher than that of the old one. Finally we describe an implementation of the new method and the traditional one (both methods are implemented in the Gapt system [12]) and show how they can be used to extract expansion proofs from proofs. We compare the implementations of the two methods and it turns out that even for very small and simple proofs a visible speed-up in computing time can be obtained.

## Preliminaries

### Sequents and Sequent Calculus

We define an extended version of Gentzen’s calculus **LK** in predicate logic with equality and arbitrary function symbols.

#### Definition 1

Let $\varGamma$ and $\varDelta$ be two multi-sets of formulas and $\vdash$ be a symbol not belonging to the logical language. Then $\varGamma \vdash \varDelta$ is called a sequent.

If $S_1:\varGamma \vdash \varDelta$ and $S_2:\varPi \vdash \varLambda$ are sequents we define the *concatenation* of $S_1$ and $S_2$ (notation $S_1 \circ S_2$) as $\varGamma ,\varPi \vdash \varDelta ,\varLambda$.

#### Definition 2

Let $S: A_{1}, \ldots , A_{n} \vdash B_{1}, \ldots , B_{m}$ be a sequent and $\mathcal {M}$ be an interpretation over the signature of $\{A_{1}, \ldots , A_{n}, B_{1}, \ldots , B_{m}\}$. Then *S* is valid in $\mathcal {M}$ if the formula $(A_{1} \wedge \ldots \wedge A_{n}) \rightarrow (B_{1} \vee \cdots \vee B_{m})$ is valid in $\mathcal {M}$. *S* is called valid if *S* is valid in all interpretations.

#### Definition 3

Let $S: A_{1}, \ldots , A_{n} \vdash B_{1}, \ldots , B_{m}$ be a sequent. *S* is called a weakly quantified sequent if there is no $\exists$ quantifier of positive polarity in some formula $A_i$ ($1 \le i \le n$) and there is no $\forall$ quantifier of positive polarity in some formula $B_j$ ($1 \le j \le m$).

#### Definition 4

(Calculus $\mathbf {LK}_=$) Basically we use Gentzen’s version of **LK** [15] but extend it by equality rules as in [2] and call the calculus $\mathbf {LK}_=$. Since we consider multi-sets of formulas, we do not need exchange or permutation rules. There are two groups of rules, the logical and the structural ones. All rules except the cut have left and right versions, denoted by *l* and *r*, respectively. The binary rules are of multiplicative type, i.e. no auto-contraction of the context is applied. In the following, *A* and *B* denote formulas whereas $\varGamma , \varDelta , \varPi , \varLambda$ denote multi-sets of formulas.

*The logical rules:* 
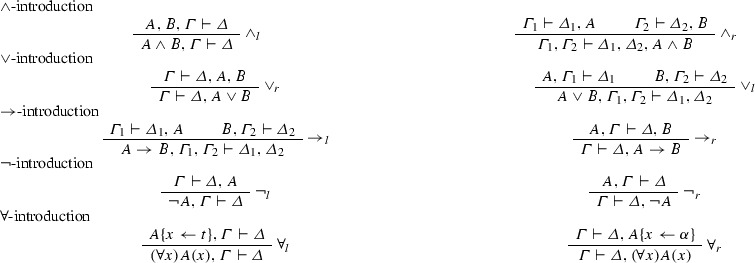


where *t* is an arbitrary term that does not contain any variables which are bound in *A* and $\alpha$ is a free variable which may not occur in $\varGamma , \varDelta , A$. $\alpha$ is called an eigenvariable.

$$\exists $$-introduction
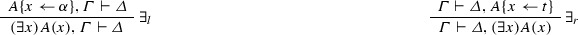


where the variable conditions for $\exists _{l}$ are the same as those for $\forall _r$ and similarly for $\exists _r$ and $\forall _l$. The quantifier-rules $\forall _l,\exists _r$ are called *weak*, the rules $\exists _l,\forall _r$*strong*.

*The structural rules:*
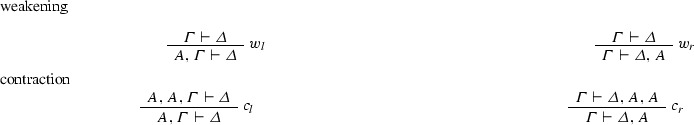
 where $$m,n \ge 1$$.

*The equality rules:*for inference on the left andon the right, where $\varLambda$ denotes a position of a subterm where replacement of *s* by *t* has to be performed. We call $s=t$ the active equation of the rules.

Note that, on atomic sequents, the rules coincide with paramodulation—under previous application of the most general unifier.

*Axioms:*

Any set of atomic sequents which is closed under substitution and contains the sequent $\vdash x=x$ (and thus all sequents of the form $\vdash t=t$ for arbitrary terms *t*) is admitted as an axiom set. We define the set $\mathrm{TAUT}= \{A \vdash A \, | \, A \text{ is } \text{ an } \text{ atom }\}$ and the axiom set $Ax = \mathrm{TAUT}\cup \{\vdash t = t \mid t \text{ a } \text{ term }\}$, which is called the standard axiom set.

An $\mathbf {LK}_=$-proof from a set of axioms $\mathcal{{A}}$ is a tree formed according to the rules of $\mathbf {LK}_=$ such that all leaves are in $\mathcal{{A}}$. The formulas in $\varGamma , \varDelta , \varPi , \varLambda$ are called context formulas. The formulas in the upper sequents that are not context formulas are called auxiliary formulas and those in the lower sequents are called main formulas. The auxiliary formulas of a cut-rule are also called cut-formulas. If $\mathcal {S}$ is a set of sequents, then an **LK**-refutation of $\mathcal {S}$ is an **LK**-tree $\pi$ where the end-sequent of $\pi$ is the empty sequent and the leaves of $\pi$ are either axioms of the standard axiom set or sequents in $\mathcal {S}$.

For the proof transformations in this paper we need the concept of ancestors of nodes in a proof tree and formula occurrences within sequents occurring in proofs.

#### Definition 5

(*Formula ancestor*) Let $\nu$ be a formula occurrence in a sequent calculus proof $\varphi$. Then $\nu$ is an ancestor of itself in $\varphi$ (the relation is reflexive). If $\nu$ is a principal formula occurrence of an inference then the occurrences of the auxiliary formula (formulas) in the premises are formula ancestors of $\nu$. If $\nu$ is not a principal occurrence then the corresponding occurrences in contexts of the (premise) premises are formula ancestors of $\nu$. The formula ancestor relation is then defined as the transitive closure.

#### Definition 6

(*Sequent ancestor*) Let $\nu$ be an occurrence of a sequent in a sequent calculus proof $\varphi$. Then $\nu$ is a sequent ancestor of itself (reflexivity). If $\nu$ corresponds to the conclusion of an inference with premises $\mu _1,\mu _2$ ($\mu$) then $\mu _1,\mu _2$ ($\mu$) are sequent ancestors of $\nu$ in $\varphi$. The sequent ancestor relation is then defined as the transitive closure.

#### Definition 7

(*Clause*) A sequent $\varGamma \vdash \varDelta$ is called a clause if $\varGamma$ and $\varDelta$ are multisets of atoms.

#### Definition 8

(*PR-calculus*) The PR-calculus works on clauses and consists of the following rules:

1. the resolution rule:  Where $n,m \ge 1$ and $\sigma$ is a most general unifier of $\{A_1,\ldots ,A_m,A'_1,\ldots ,A'_n\}$. It is also required that $\varGamma \vdash \varDelta , A$ and $\varGamma ', A' \vdash \varDelta '$ are variable disjoint.
2. the paramodulation rules:
  We assume that the two clauses in the premises are always variable disjoint and that $\sigma$ is a most general unifier of $\{s,s'\}$.  for inference on the left side of the clauses and  for the right side, where $\varLambda$ denotes a position of a subterm where $s'$ is replaced by *t*. We call $s=t$ the *active equation* of the rules.

A *PR-derivation* from a set of clauses $\mathcal{{C}}$ is a tree derivation based on the rules above where all clauses in the leaves are variants of clauses in $\mathcal{{C}}$. A *PR-derivation* of $\vdash$ from $\mathcal{{C}}$ is called a *PR-refutation* of $\mathcal{{C}}$.

#### Definition 9

Let $\varphi$ be a proof and $\eta$ be some arbitrary inference in $\varphi$. We say that $\eta$ goes into the end-sequent of $\varphi$ if the principal formula of $\eta$ is an ancestor of the end-sequent. In this case, $\eta$ cannot be a cut. $\eta$ goes into a cut otherwise.

### Expansion Trees

Expansion trees, first introduced in [23], are natural structures representing the instantiated variables for quantified formulas.

These structures record the substitutions for quantifiers in the original formula and the formulas resulting from instantiations. Expansion trees may contain logical connectives as well as the new connective $+^{t}$, where *t* is a term. Informally, an expression of the kind $Q x A(x) +^{t_{1}} E_{1} +^{t_2} \cdots +^{t_n} E_{n}$ is an expansion tree, where $Q \in \{\forall , \exists \}$ and $t_{1},\ldots ,t_{n}$ are terms such that this expansion tree represents the result when instantiating the quantified expression *QxA*(*x*) with the terms $t_{1},\ldots ,t_{n}$ to get the structures $E_i$. $E_i$ is again an expansion tree representing $A(t_{i})$ for $i = 1,\ldots ,n$.

Our definition is a modified one as our proofs are skolemized and we do not have quantifiers with eigenvariable conditions. The definition below takes care that only trees with weak quantifiers are constructed.

#### Definition 10

Expansion trees, dual expansion trees and a function ${ Sh}$ (shallow) which maps expansion trees to formulas are defined inductively as follows:

1. If *A* is a quantifier-free formula then *A* is an expansion tree (and a dual expansion tree) for *A* and $Sh(A) = A$.
2. If *E* is an expansion tree then $\lnot E$ is a dual expansion tree and ${ Sh}(\lnot E) = \lnot { Sh}(E)$.
3. If *E* is a dual expansion tree then $\lnot E$ is an expansion tree and ${ Sh}(\lnot E) = \lnot { Sh}(E)$.
4. If $E_1$ and $E_2$ are (dual) expansion trees, then $E_{1} \wedge E_2$, $E_{1} \vee E_2$ are (dual) expansion trees and ${ Sh}(E_{1} \wedge E_{2}) = { Sh}(E_{1}) \wedge { Sh}(E_{2})$, the same for $\vee$.
5. If $E_1$ is a dual expansion tree and $E_2$ is an expansion tree then $E_1 \rightarrow E_2$ is an expansion tree and ${ Sh}(E_1 \rightarrow E_2) = { Sh}(E_1) \rightarrow { Sh}(E_2)$.
6. If $E_1$ is an expansion tree and $E_2$ is a dual expansion tree then $E_1 \rightarrow E_2$ is a dual expansion tree and ${ Sh}(E_1 \rightarrow E_2) = { Sh}(E_1) \rightarrow { Sh}(E_2)$.
7. Let *A*(*x*) be a formula and $t_{1},\ldots ,t_{n}$ ($n \ge 1$) be a list of terms. Let $E_{1}, \ldots , E_{n}$ be expansion trees with ${ Sh}(E_{i}) = A(t_{i})$ for $i = 1,\ldots ,n$; then $\exists x A(x) +^{t_1} E_{1} +^{t_2} \cdots +^{t_n} E_{n}$ is an expansion tree with ${ Sh}(\exists x A(x) +^{t_1} E_{1} +^{t_2} \cdots +^{t_n} E_{n}) = \exists x A(x)$.
8. Let *A*(*x*) be a formula and $t_{1},\ldots ,t_{n}$ ($n \ge 1$) be a list of terms. Let $E_{1}, \ldots , E_{n}$ be dual expansion trees with ${ Sh}(E_{i}) = A(t_{i})$ for $i = 1,\ldots ,n$; then $\forall x A(x) +^{t_1} E_{1} +^{t_2} \cdots +^{t_n} E_{n}$ is a dual expansion tree with ${ Sh}(\forall x A(x) +^{t_1} E_{1} +^{t_2} \cdots +^{t_n} E_{n}) = \forall x A(x)$.

#### Example 1

Let *P*(*x*) be an atom. Then *P*(*a*) is a dual expansion tree and $\forall x.P(x) +^a P(a)$ is a dual expansion tree. $\exists x.P(x) +^a P(a)$ is an expansion tree. So

$$\begin{aligned} \forall x.P(x) +^a P(a) \rightarrow \exists x.P(x) +^a P(a) \end{aligned}$$

is an expansion tree. But note that

$$\begin{aligned} \forall x.P(x) +^a P(a) \rightarrow \forall x.P(x) +^a P(a) \end{aligned}$$

is *not* an expansion tree according to Definition 10 as $\forall x.P(x) +^a P(a)$ is a dual expansion tree but not an expansion tree. Indeed, having strong quantifiers with the type of expansion defined above would be unsound.

The function ${ Dp}$ (deep) maps expansion trees (and dual expansion trees) to quantifier-free formulas, their *full expansion*.

#### Definition 11

${ Dp}$ maps a (dual) expansion tree to a formula as follows:

$$\begin{aligned} \begin{array}{lcl} { Dp}(E) &{} = &{} E \text { for an atomic expansion tree E}, \\ { Dp}(\lnot E) &{} = &{} \lnot { Dp}(E), \\ { Dp}(E_{1} \circ E_{2}) &{} = &{} { Dp}(E_{1}) \circ { Dp}(E_{2}) \text { for } \circ \in \{\wedge , \vee , \rightarrow \}, \\ { Dp}(\exists x A +^{t_{1}} E_{1} +^{t_{2}} \cdots +^{t_{n}} E_{n}) &{} = &{} { Dp}(E_{1}) \vee \cdots \vee { Dp}(E_{n}), \\ { Dp}(\forall x A +^{t_{1}} E_{1} +^{t_{2}} \cdots +^{t_{n}} E_{n}) &{} = &{} { Dp}(E_{1}) \wedge \cdots \wedge { Dp}(E_{n}). \end{array} \end{aligned}$$

In [23] a notion of expansion proof was defined from expansion trees using the conditions acyclicity and tautology. Acyclicity ensures that there are no cycles between the strong quantifier nodes in the expansion tree. Since our formulas are skolemized and hence do not contain strong quantifiers, we do not need this condition.

#### Definition 12

(*Expansion proof*) Let *ET* be an expansion tree of a formula *A* without strong quantifiers. Then *ET* is called an expansion proof of *A* from a set of axioms $\mathcal{{A}}$ if $Sh(ET) = A$ and $\mathcal{{A}}\models { Dp}(ET)$ (where $\models$ is the consequence relation in predicate logic with equality).

Expansion proofs encode a proof of validity of the formula they represent. They can be directly translated into sequent calculus, see [23], and the transformation is based on so-called *q*-sequents, which we refer to as s-expansion trees (sequent of expansion trees) in this paper.

#### Definition 13

(*S-expansion tree*) The structure $S:\ \varGamma \vdash \varDelta$ where $\varDelta :Q_{1}, \ldots , Q_{s}$ is a multiset of expansion trees, $\varGamma :P_{1}, \ldots , P_{r}$ is a multiset of dual expansion trees is called an *s-expansion tree*. If $\lnot \varGamma \vee \varDelta$ (which stands for $\lnot P_{1} \vee \cdots \vee \lnot P_{r} \vee Q_{1} \vee \cdots \vee Q_{s}$) is an expansion proof then *S* is called an *s-expansion proof*. This expansion proof is the expansion proof associated with *S*; the sequent

$$\begin{aligned} { Seq}(S):\ Sh(P_{1}),\ldots ,Sh(P_{r}) \rightarrow Sh(Q_{1}), \ldots , Sh(Q_{s}) \end{aligned}$$

is the sequent associated with *S*.

It is also possible to read off expansion proofs from sequent calculus proofs. Note that the expansion proof of a proof $\varphi$ is a sequent of expansion trees, which are defined to be the expansion trees of all formulas in the end-sequent of $\varphi$. An algorithm for the extraction of expansion proofs from sequent calculus proofs is presented in [23] and modified algorithms (dealing with cuts and equality) are presented in [21, 22]. There exist also algorithms for a transformation of resolution-trees into expansion-trees, see [24].

We will use an algorithm that is briefly described in [21]. In order to show how an expansion proof is extracted from a proof in $\mathbf {LK}_=$, we first need to define an operation on expansion trees. The Merge operator on expansion trees is defined in [23]. Intuitively, two expansion trees $T_1$ and $T_2$ can be merged, if $Sh(T_1) = Sh(T_2 )$. We give a definition adapted to our concept of expansion tree.

#### Definition 14

(*Merge*) Let $E_1,E_2$ be (dual) expansion trees such that ${ Sh}(E_1) = { Sh}(E_2)$. We define the merge inductively on the complexity of $E_1$.

- If $$E_1$$ is an atom then $$E_2$$ is an atom too and $$E_1=E_2$$; we define $$\mathrm{Merge}_t(E_1,$$$$E_2) = E_1$$.
- If $E_1 = \lnot E'_1$. Then $E_2 = \lnot E'_2$ for some $E'_2$. Let $\mathrm{Merge}_t(E'_1,E'_2) = E'_3$, then $\mathrm{Merge}_t(E_1,E_2) = \lnot E'_3$.
- Let $E_1 = E_{11} \circ E_{12}$ for $\circ \in \{\wedge ,\vee ,\rightarrow \}$. Then $E_2 = E_{21} \circ E_{22}$ for some $E_{21},E_{22}$. Let $E'_1 = \mathrm{Merge}_t(E_{11},E_{21})$ and $E'_2 = \mathrm{Merge}_t(E_{21},E_{22})$. Then $\mathrm{Merge}_t(E_1,E_2) = E'_1 \circ E'_2$.
- Let $E_1 = Q x.A(x) +^{t_1} E_{11} + \cdots +^{t_n} E_{1n}$. Then $E_2$ is of the form $Q x.A(x) +^{s_1} E_{21} + \cdots +^{s_m} E_{2m}$. Then
  $$\begin{aligned} \mathrm{Merge}_t(E_1,E_2) = Q x.A(x) +^{t_1} E_{11} + \cdots +^{t_n} E_{1n} +^{s_1} E_{21} + \cdots +^{s_m} E_{2m}. \end{aligned}$$

#### Example 2

Let $T_1 = \forall x Px +^{a} Pa$ and $T_2 = \forall x Px +^b Pb$ be two dual expansion trees then

$$\begin{aligned} \mathrm{Merge}_t(T_1,T_2) = \forall x Px +^{a} Pa +^b Pb. \end{aligned}$$

The definition can be easily extended to more than two expansion trees. Let $T_1 , \ldots , T_n$ (for $n \ge 2$) be (dual) expansion trees such that ${ Sh}(T_i) = { Sh}(T_j)$ for all $i,j \in \{1,\ldots ,n\}$. Then we define

$$\begin{aligned} \mathrm{merge}_t(T_1,T_2)= & {} \mathrm{Merge}_t(T_1,T_2),\\ \mathrm{merge}_t(T_1,\ldots ,T_n)= & {} \mathrm{Merge}_t(\mathrm{merge}_t(T_1,\ldots ,T_{n-1}),T_n) \text{ for } n>2. \end{aligned}$$

It is also possible to merge s-expansion trees. As an *s*-expansion tree is defined via multisets of expansion trees, some expansion trees might occur more than once either on the left or on the right. In such cases merging *s*-expansion trees might become ambiguous. To avoid this ambiguity we restrict the merge of *s*-expansion trees to so-called normalized ones, where the shallow forms occur only once.

#### Definition 15

(*Normalized sequents*) A sequent $\varGamma \vdash \varDelta$ is called *normalized* if the multiplicity of all formulas occurring in $\varGamma$ ($\varDelta$) is one, more precisely: if $\varGamma = A_1,\ldots ,A_n$ and $\varDelta = B_1,\ldots ,B_m$ then $A_i \ne A_j$ for $i \ne j$ ($i,j \in \{1,\ldots ,n\}$) and $B_l \ne B_k$ for $l \ne k$ ($l,k \in \{1,\ldots ,m\}$). Let *S* be an *s*-expansion tree then *S* is called normalized if ${ Seq}(S)$ is normalized.

#### Remark 1

Note that every **LK**-proof $\varphi$ of *S* and every *s*-expansion tree can be easily transformed into a proof (*s*-expansion tree) of a normalized sequent: just apply the rules $c_l,c_r$ to *S*. In Sect. 4 we will merge *s*-expansion trees only if they correspond to end-sequents of proofs. Therefore restricting the merge to normalized *s*-expansion proofs does not affect the generality of our approach.

In normalized sequents the multisets become sets which allows us to define some set-based operations on sequents:

#### Definition 16

Let $S_1,S_2$ be two normalized sequents such that $S_1 = \varGamma _1 \vdash \varDelta _1$, $S_2 = \varGamma _2 \vdash \varDelta _2$. Let $\varGamma = \varGamma _1 \cap \varGamma _2$, $\varDelta = \varDelta _1 \cap \varDelta _2$. We define the following operations on $S_1,S_2$:

$$\begin{aligned} S_1 \cap S_2 = \varGamma \vdash \varDelta ,\ S_1 \setminus S_2 = (\varGamma _1 \setminus \varGamma ) \vdash (\varDelta _1 \setminus \varDelta ),\ S_2 \setminus S_1 = (\varGamma _2 \setminus \varGamma ) \vdash (\varDelta _2 \setminus \varDelta ). \end{aligned}$$

The sequents $S_1$ and $S_2$ are called *disjoint* if $S_1 \cap S_2 =\ \vdash$.Then, obviously, $S_1 \cap S_2$, $S_1 \setminus S_2$ and $S_2 \setminus S_1$ are pairwise disjoint and

$$\begin{aligned} S_1= & {} (S_1 \cap S_2) \circ (S_1 \setminus S_2),\\ S_2= & {} (S_1 \cap S_2) \circ (S_2 \setminus S_1). \end{aligned}$$

Now we are ready to define the merging of normalized *s*-expansion trees.

#### Definition 17

(*Merge ofs*-*expansion trees*) Let $S_1$ and $S_2$ be two normalized *s*-expansion trees and $S^*_1 = { Seq}(S_1)$, $S^*_2 = { Seq}(S_2)$. Then, by definition, $S^*_1,S^*_2$ are normalized sequents. We define $\varGamma ^* \vdash \varDelta ^* = S^*_1 \cap S^*_2$, $\varPi ^*_1 \vdash \varLambda ^*_1 = S^*_1 \setminus S^*_2$, $\varPi ^*_2 \vdash \varLambda ^*_2 = S^*_2 \setminus S^*_1$. Then

$$\begin{aligned} S^*_1= & {} (\varGamma ^* \vdash \varDelta ^*) \circ (\varPi ^*_1 \vdash \varLambda ^*_1),\\ S^*_2= & {} (\varGamma ^* \vdash \varDelta ^*) \circ (\varPi ^*_2 \vdash \varLambda ^*_2). \end{aligned}$$

Then there exist *s*-expansion trees $\varGamma \vdash \varDelta$, $\varGamma ' \vdash \varDelta '$, $\varPi _1 \vdash \varLambda _1$ and $\varPi _2 \vdash \varLambda _2$ such that

$$\begin{aligned} S_1= & {} (\varGamma \vdash \varDelta ) \circ (\varPi _1 \vdash \varLambda _1),\\ S_2= & {} (\varGamma ' \vdash \varDelta ') \circ (\varPi _2 \vdash \varLambda _2), \end{aligned}$$

where ${ Seq}(\varGamma \vdash \varDelta ) = { Seq}(\varGamma ' \vdash \varDelta ') = \varGamma ^* \vdash \varDelta ^*$, ${ Seq}(\varPi _1 \vdash \varLambda _1) = \varPi ^*_1 \vdash \varLambda ^*_1$ and ${ Seq}(\varPi _2 \vdash \varLambda _2) = \varPi ^*_2 \vdash \varLambda ^*_2$. Note that the concatenation $\circ$ of sequents can be directly extended to *s*-expansion trees.

Then there exist bijective mappings $\pi _l:\varGamma \rightarrow \varGamma '$ and $\pi _r:\varDelta \rightarrow \varDelta '$ with $\pi _l(T) = T'$ iff ${ Sh}(T) = { Sh}(T')$ (the same for $\pi _r$). So assume

$$\begin{aligned} \varGamma \vdash \varDelta= & {} T_1,\ldots ,T_n \vdash T_{n+1},\ldots ,T_{n+m} \text{ and } \text{ therefore } \\ \varGamma ' \vdash \varDelta '= & {} \pi _l(T_1),\ldots ,\pi _l(T_n) \vdash \pi _r(T_{n+1}),\ldots ,\pi _r(T_{n+m}). \end{aligned}$$

Now let $T^*_i = \mathrm{merge}_t(T_i,\pi _l(T_i))$ for $i=1,\ldots ,n$ and $T^*_i = \mathrm{merge}_t(T_i,\pi _r(T_i))$ for $i=n+1,\ldots ,n+m$. Then we define

$$\begin{aligned} \mathrm{Merge}_s(S_1,S_2) = (T^*_1,\ldots ,T^*_n \vdash T^*_{n+1},\ldots ,T^*_{n+m})\circ (\varPi _1 \vdash \varLambda _1) \circ (\varPi _2 \vdash \varLambda _2). \end{aligned}$$

Note that, by construction, $\mathrm{Merge}_s(S_1,S_2)$ is a normalized *s*-expansion tree.

We extend the merging of *s*-sequents to more than two as follows. Let $n \ge 2$ and $S_1,\ldots ,S_n$ be normalized *s*-expansion trees. Then

$$\begin{aligned} \mathrm{merge}_s(S_1,S_2)= & {} \mathrm{Merge}_s(S_1,S_2) \text{ for } n=2,\\ \mathrm{merge}_s(S_1,\ldots ,S_n)= & {} \mathrm{Merge}_s(\mathrm{merge}_s(S_1,\ldots ,S_{n-1}),S_n) \text{ for } n>2. \end{aligned}$$

The *s*-expansion tree $\mathrm{merge}_s(S_1,\ldots ,S_n)$ is also normal which can be verified by an obvious inductive argument.

Frequently we will write $\mathrm{merge}_s\{S_i \mid i=1,\ldots ,n\}$ for $\mathrm{merge}_s(S_1,\ldots ,S_n)$. If no confusion arises we will frequently write $\mathrm{merge}$ instead of $\mathrm{merge}_t$ and $\mathrm{merge}_s$.

#### Example 3

Let $S_1 = \forall x Px +^a Pa \vdash$, $S_2 = \forall x Px +^b Pb \vdash Qa$ and $S_3 = \ \vdash \exists y Qy$ be s-expansion trees. Then

$$\begin{aligned} \mathrm{merge}_s(S_1, S_2 , S_3) = \forall x Px +^a Pa +^b Pb \vdash Qa, \exists y Qy. \end{aligned}$$

The extraction of expansion proofs from **LK**-proofs requires quantifier-free cuts. Due to the structure of the CERES-method (which will be used for a efficient method of extracting expansion proofs) we consider proofs with only atomic cuts.

#### Definition 18

A proof $\varphi$ in $\mathbf {LK}_=$ is in the subclass $\mathbf {LK}_0$ if

1. $\varphi$ does not contain strong quantifier inferences.
2. All cuts in $\varphi$ are atomic.
3. Equality rules are only applied to atoms.
4. The axiom set contains *Ax*.

#### Definition 19

(*Extraction ofs-expansion trees from proofs in*$\mathbf {LK}_0$) We define a transformation $\mathrm{ET}$ which maps proofs in $\mathbf {LK}_0$ to *s*-expansion trees. We define the transformation inductively (on the number of inferences in the proof) but the rules for $\lnot _l , \lnot _r , \vee _l , \vee _{r_1}, \vee _{r_2}, =_{l2}, =_{r2}$ are omitted, the transformation of the these rules being obvious.

base case: $\varphi$ is an axiom. Then $\varphi$ is of the form $A_1,\ldots ,A_n \vdash B_1,\ldots ,B_m$ for atoms $A_i,B_j$ and so $\mathrm{ET}(\varphi ) = \varphi$.

If $$\varphi = $$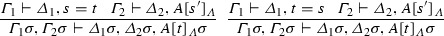 and $$\mathrm{ET}(\pi ) = A^{*},B^*, \varGamma ^{*} \vdash \varDelta ^{*}$$, then $$\mathrm{ET}(\varphi ) = A^{*} \wedge B^*, \varGamma ^{*} \vdash \varDelta ^{*}$$.

If $$\varphi = $$
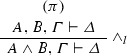
 and $$\mathrm{ET}(\pi _1 ) = \varGamma _{1}^{*}$$$$\vdash $$$$\varDelta _{1}^{*}, A^{*}$$ and $$\mathrm{ET}(\pi _2 ) = \varGamma _{2}^{*}$$$$\vdash $$$$\varDelta _{2}^{*}, B^{*}$$, then $$\mathrm{ET}(\varphi ) = \varGamma _{1}^{*},$$$$\varGamma _{2}^{*}$$$$\vdash $$$$\varDelta _{1}^{*},$$$$\varDelta _{2}^{*},$$$$A^{*} \wedge B^{*}$$.

If $$\varphi = $$
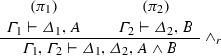
 and $\mathrm{ET}(\pi _1 ) = \varGamma _{1}^{*} \vdash \varDelta _{1}^{*}, A^{*}$ and $\mathrm{ET}(\pi _2 ) = B^{*}, \varGamma _{2}^{*} \vdash \varDelta _{2}^{*}$, then $\mathrm{ET}(\varphi ) = A^{*} \rightarrow B^{*}, \varGamma _{1}^{*}, \varGamma _{2}^{*} \vdash \varDelta _{1}^{*}, \varDelta _{2}^{*}$.

If $$\varphi = $$
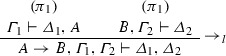
 and $$\mathrm{ET}(\pi ) = A^{*}, \varGamma ^{*} \vdash \varDelta ^{*}, B^{*}$$, then $$\mathrm{ET}(\varphi ) = \varGamma ^{*} \vdash \varDelta ^{*}, A^{*} \rightarrow B^{*}$$.

If $$\varphi = $$
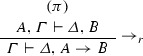
 and $\mathrm{ET}(\pi ) = A\{x \leftarrow t\}^{*}, \varGamma ^{*} \vdash \varDelta ^{*}$, then $\mathrm{ET}(\varphi ) = (\forall x) A(x) +^{t} A\{x \leftarrow t\}^{*}, \varGamma ^{*} \vdash \varDelta ^{*}$. If $\varphi =$
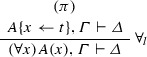
 and $\mathrm{ET}(\pi ) = \varGamma ^{*} \vdash \varDelta ^{*}, A\{x \leftarrow t\}^{*}$, then $\mathrm{ET}(\varphi ) = \varGamma ^{*} \vdash \varDelta ^{*}, (\exists x) A(x) +^{t} A\{x \leftarrow t\}^{*}$. If $\varphi =$
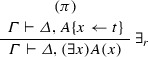
 and $\mathrm{ET}(\pi ) = \varGamma ^{*} \vdash \varDelta ^{*}$, then $\mathrm{ET}(\varphi ) = A, \varGamma ^{*} \vdash \varDelta ^{*}$. Similarly for $w_r$.

If $$\varphi = $$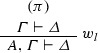 and $\mathrm{ET}(\pi ) = A_{1}^{*}, A_{2}^{*}, \varGamma ^{*} \vdash \varDelta ^{*}$ then $\mathrm{ET}(\varphi ) = {\mathrm{merge}}(A_{1}^{*}, A_{2}^{*}), \varGamma ^{*} \vdash \varDelta ^{*}$. Similarly for $c_r$.

Note that, for the rules below, the auxiliary formulas of the rules are atomic.

If $$\varphi = $$
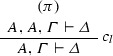
 where $\mathrm{ET}(\pi _1 ) = \varGamma _{1}^{*} \vdash \varDelta _{1}^{*}, A^{m}$ and $\mathrm{ET}(\pi _1 ) = A^{n}, \varGamma _{2}^{*} \vdash \varDelta _{2}^{*}$; then $\mathrm{ET}(\varphi ) = \varGamma _{1}^{*}, \varGamma _{2}^{*} \vdash \varDelta _{1}^{*}, \varDelta _{2}^{*}$.

If $$\varphi = $$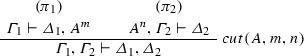 and $\mathrm{ET}(\pi _1 ) = \varGamma _{1}^{*} \vdash \varDelta _{1}^{*}, s=t$ and $\mathrm{ET}(\pi _2 ) = A[s]_{\varLambda }, \varGamma _{2}^{*} \vdash \varDelta _{2}^{*}$, then $\mathrm{ET}(\varphi ) = \varGamma _{1}^{*}, \varGamma _{2}^{*}, A[t]_{\varLambda } \vdash \varDelta _{1}^{*}, \varDelta _{2}^{*}$.

If $$\varphi = $$
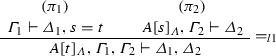
 and $\mathrm{ET}(\pi _1 ) = \varGamma _{1}^{*} \vdash \varDelta _{1}^{*}, s=t$ and $\mathrm{ET}(\pi _1 ) = \varGamma _{2}^{*} \vdash \varDelta _{2}^{*}, A[s]_{\varLambda }$, then $\mathrm{ET}(\varphi ) = \varGamma _{1}^{*}, \varGamma _{2}^{*} \vdash \varDelta _{1}^{*}, \varDelta _{2}^{*}, A[t]_{\varLambda }$.

#### Proposition 1

The transformation $\mathrm{ET}$ is sound: if $\varphi$ is a proof in $\mathbf {LK}_0$ then $\mathrm{ET}(\varphi )$ is an *s*-expansion proof.

#### Proof

We proceed by induction on the number of inferences in $\varphi$. We consider the cases of axioms (represented by an axiom set $\mathcal{{A}}$), $\wedge _r$, *cut* and $=_{r_1}$; the other cases are analogous.

- (axiom) Let *S* be an axiom sequent in $\mathcal{{A}}$. Then $S = A_1,\ldots ,A_n \vdash B_1,\ldots ,B_m$ for atoms $A_i,B_j$. Therefore for $F_S:\lnot A_1 \vee \cdots \vee \lnot A_n \vee B_1 \vee \cdots B_m$ we have ${ Sh}(F_S) = { Dp}(F_S) = F_S$ and $\mathcal{{A}}\models { Dp}(F_S)$.
- ($$\wedge _r$$) $$\varphi = $$
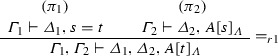
 and $$\mathrm{ET}(\pi _1 ) = \varGamma _{1}^{*}$$$$\vdash $$$$\varDelta _{1}^{*}, A^{*}$$ and $$\mathrm{ET}(\pi _2 ) = \varGamma _{2}^{*}$$$$\vdash $$$$\varDelta _{2}^{*}, B^{*}$$ are *s*-expansion-proofs. Therefore, $$\lnot \varGamma _{1}^{*} \vee \varDelta _{1}^{*} \vee A^{*}$$ and $$\lnot \varGamma _{2}^{*} \vee \varDelta _{2}^{*} \vee B^{*}$$ are expansion proofs and $$\mathcal{{A}}\models { Dp}(\lnot \varGamma _{1}^{*} \vee \varDelta _{1}^{*} \vee A^{*})$$ and $$\mathcal{{A}}\models { Dp}(\lnot \varGamma _{2}^{*} \vee \varDelta _{2}^{*} \vee B^{*})$$. But then $$\mathcal{{A}}\models { Dp}(\lnot \varGamma _{1}^{*} \vee \varDelta _{1}^{*} \vee \lnot \varGamma _{2}^{*} \vee \varDelta _{2}^{*} \vee (A^{*} \wedge B^{*}))$$ and $$\lnot \varGamma _{1}^{*} \vee \varDelta _{1}^{*} \vee \lnot \varGamma _{2}^{*} \vee \varDelta _{2}^{*} \vee (A^{*} \wedge B^{*})$$ is an expansion proof. Therefore $$\varGamma _{1}^{*}, \varGamma _{2}^{*} \vdash \varDelta _{1}^{*}, \varDelta _{2}^{*}, A^{*} \wedge B^{*}$$ ($$= \mathrm{ET}(\varphi )$$) is an s-expansion-proof.
- (*cut*) $$\varphi = $$
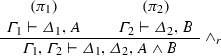
 where $\mathrm{ET}(\pi _1 ) = \varGamma _{1}^{*} \vdash \varDelta _{1}^{*}, A^{m}$ and $\mathrm{ET}(\pi _1 ) = A^{n}, \varGamma _{2}^{*} \vdash \varDelta _{2}^{*}$ are s-expansion-proofs. Therefore, $\lnot \varGamma _{1}^{*} \vee \varDelta _{1}^{*} \vee A^{m}$ and $\lnot A^{n} \vee \lnot \varGamma _{2}^{*} \vee \varDelta _{2}^{*}$ are expansion proofs and $\mathcal{{A}}\models { Dp}(\lnot \varGamma _{1}^{*} \vee \varDelta _{1}^{*} \vee A^{m})$ and $\mathcal{{A}}\models { Dp}(\lnot A^{n} \vee \lnot \varGamma _{2}^{*} \vee \varDelta _{2}^{*})$. But then $\mathcal{{A}}\models { Dp}(\lnot \varGamma _{1}^{*} \vee \varDelta _{1}^{*} \vee \lnot \varGamma _{2}^{*} \vee \varDelta _{2}^{*})$ and $\lnot \varGamma _{1}^{*} \vee \varDelta _{1}^{*} \vee \lnot \varGamma _{2}^{*} \vee \varDelta _{2}^{*}$ is an expansion proof. Therefore $\varGamma _{1}^{*}, \varGamma _{2}^{*} \vdash \varDelta _{1}^{*}, \varDelta _{2}^{*}$ ($= \mathrm{ET}(\varphi )$) is an s-expansion-proof.
- ($$=_{r_1}$$) $$\varphi = $$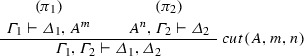 and $\mathrm{ET}(\pi _1 ) = \varGamma _{1}^{*} \vdash \varDelta _{1}^{*}, s=t$ and $\mathrm{ET}(\pi _1 ) = \varGamma _{2}^{*} \vdash \varDelta _{2}^{*}, A[s]_{\varLambda }$ are s-expansion proofs. Therefore $\lnot \varGamma _{1}^{*} \vee \varDelta _{1}^{*} \vee s=t$ and $\lnot \varGamma _{2}^{*} \vee \varDelta _{2}^{*} \vee A[s]_{\varLambda }$ are expansion proofs and hence $\mathcal{{A}}\models { Dp}(\lnot \varGamma _{1}^{*} \vee \varDelta _{1}^{*} \vee s=t)$ and $\mathcal{{A}}\models { Dp}(\lnot \varGamma _{2}^{*} \vee \varDelta _{2}^{*} \vee A[s]_{\varLambda })$. But then $\mathcal{{A}}\models { Dp}(\lnot \varGamma _{1}^{*} \vee \varDelta _{1}^{*} \vee \lnot \varGamma _{2}^{*} \vee \varDelta _{2}^{*} \vee A[t]_{\varLambda })$ and $\lnot \varGamma _{1}^{*} \vee \varDelta _{1}^{*} \vee \lnot \varGamma _{2}^{*} \vee \varDelta _{2}^{*} \vee A[t]_{\varLambda })$ is an expansion proof. Therefore, $\varGamma _{1}^{*}, \varGamma _{2}^{*} \vdash \varDelta _{1}^{*}, \varDelta _{2}^{*}, A[t]_{\varLambda }$ ($= \mathrm{ET}(\varphi )$) is an s-expansion-proof. $\square$

In case of a prenex end-sequent *S* an expansion proof corresponds to the derivation of *S* from the mid-sequent (Herbrand sequent). The essence of Herbrand’s theorem [18] consists of the replacement of quantified formulas by instances of these formulas. This results in a quantifier-free formula which is validity-equivalent to the original formula. Of course, the function ${ Dp}$ of an expansion proof corresponds to a Herbrand sequent. But if we consider proofs of prenex end-sequents we can extract Herbrand sequents directly, instead of extracting expansion proofs and computing their deep functions. The method for Herbrand sequent extraction in the prenex case is based on collecting instances, and is described in [6, 20].

To illustrate construction of an *s*-expansion proof from an $\mathbf {LK}_0$-proof , consider the following simple example.

#### Example 4

We work with $Ax \cup \{\vdash a=b\}$. Let $\varphi$ be the proof of $S = P(a) \wedge Q(a, f(a)) \vdash \exists x (P(x) \wedge \exists y. Q(x, y))$: 
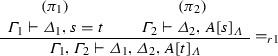


Note that *S* is not in prenex form. Therefore, extracting the Herbrand sequent by collecting instances is not possible. Instead we compute the expansion proof $\mathrm{ET}(\varphi )$.

Below we compute the *s*-expansion proof corresponding to $\varphi$. First we compute expansion trees for all formulas *F* in *S* and call them $\mathrm{ET}(F)$.

$$\begin{aligned} \begin{array}{lcl} \mathrm{ET}(P(a) \wedge Q(a, f(a))) &{} = &{} P(a) \wedge Q(a, f(a)) \\ \mathrm{ET}(\exists x (P(x) \wedge \exists y. Q(x, y))) &{} = &{} \exists x (P(x) \wedge \exists y. Q(x, y)) \\ &{} &{} +^{a} (P(a) \wedge (\exists y Q(a,y) +^{f(b)} Q(a, f(b)))) \end{array} \end{aligned}$$

The *s*-expansion proof $\mathrm{ET}(\varphi )$ associated with the end-sequent *S* is:

$$\begin{aligned} \begin{array}{lcl} \mathrm{ET}(P(a) \wedge Q(a, f(a)))&\vdash&\mathrm{ET}(\exists x (P(x) \wedge \exists y. Q(x, y))) \end{array} \end{aligned}$$

The corresponding expansion proof associated with $\mathrm{ET}(\varphi )$ is:

$$\begin{aligned} \lnot \mathrm{ET}(P(a) \wedge Q(a, f(a))) \vee \mathrm{ET}(\exists x (P(x) \wedge \exists y. Q(x, y))). \end{aligned}$$

To obtain the tautologous formula (corresponding to the Herbrand sequent) we construct the deep function for the expansion proof; we compute ${ Dp}(\mathrm{ET}(S_{i}))$:

$$\begin{aligned}&\begin{array}{lclcl} { Dp}(\mathrm{ET}(P(a) \wedge Q(a, f(a))))= & {} { Dp}(P(a)) \wedge { Dp}(Q(a, f(a)))= & {} P(a) \wedge Q(a, f(a)) \end{array} \\&\quad \begin{array}{lcl} { Dp}(\mathrm{ET}(\exists x (P(x) \wedge \exists y. Q(x, y)))) &{} = &{} \\ { Dp}(\exists x (P(x) \wedge \exists y. Q(x, y)) +^{a} (P(a) \wedge (\exists y Q(a,y) +^{f(b)} Q(a, f(b))))) &{} = &{} \\ { Dp}(P(a) \wedge (\exists y Q(a,y) +^{f(b)} Q(a, f(b)))) &{} = &{} \\ { Dp}(P(a)) \wedge { Dp}(\exists y Q(a,y) +^{f(b)} Q(a, f(b))) &{} = &{} \\ P(a) \wedge Q(a, f(b)) &{} &{} \end{array} \end{aligned}$$

Hence, combining those deep functions we get $P(a) \wedge Q(a, f(a)) \vdash P(a) \wedge Q(a, f(b))$. Note that this sequent is valid in $Ax \cup \{\vdash a=b\}$ (though it is not tautological).

## The Method CERES

The method CERES [4, 5], is a cut-elimination method that is based on resolution. It differs from the reductive stepwise methods a la Gentzen [15] by analyzing the whole proof in a preprocessing step and extracting a formula in clausal form which forms the kernel of the cut-elimination method.

CERES in predicate logic with equality roughly works as follows: The structure of a proof $\varphi$ containing cuts is encoded in an unsatisfiable set of clauses $\mathrm{CL}(\varphi )$ (the *characteristic clause set* of $\varphi$). A refutation of $\mathrm{CL}(\varphi )$ by resolution and paramodulation (abbreviated as PR-refutation) then serves as a skeleton for an *atomic cut normal form*, a new proof which contains at most atomic cuts. The corresponding proof theoretic transformation uses so-called proof projections $\varphi [C]$ for $C \in \mathrm{CL}(\varphi )$, which are simple cut-free proofs extracted from $\varphi$ (proving the end-sequent *S* extended by the atomic sequent *C*). In [5] it was shown that CERES outperforms reductive methods of cut-elimination (a la Gentzen or Tait) in computational complexity: there are infinite sequences of proofs where the computing time of CERES is nonelementarily faster than that of the reductive methods; on the other hand a nonelementary speed-up of CERES via reductive methods is shown impossible.

In this section we describe the original CERES method which was designed for classical logic. Given an $\mathbf {LK}_=$-proof $\varphi$ of a skolemized sequent $\varGamma \vdash \varDelta$, the main steps of (classical) CERES are:

1. Extraction of the characteristic clause set $\mathrm{CL}(\varphi )$.
2. Construction of a PR-refutation (see Definition 8) of $\mathrm{CL}(\varphi )$.
3. Extraction of a set of projections $\pi (C)$ for every $C \in \mathrm{CL}(\varphi )$.
4. Merging of refutation and projections into a proof $\varphi ^*$ (a CERES-normal form) with only atomic cuts.

We will use the following proof as our running example to clarify the definitions below.

### Example 5

The set of axioms $Ax_s$ is defined as $Ax_s = Ax \cup \{ \vdash f^2 z = gz\}$.

Let $\varphi$ be a proof of the sequent $Pa, \forall x (Px \rightarrow Pfx) \vdash \exists z Pf^{4}z$. 
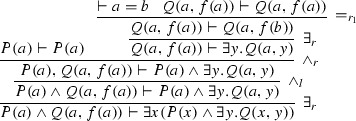
$$\varphi _1$$ is 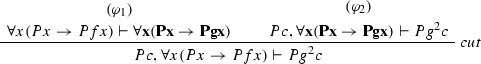
$$\varphi _2$$ is 
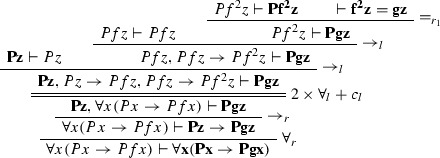
 The ancestors of the cut formulas are indicated in bold face.

Intuitively, the clause set extraction consists in collecting all atomic ancestors of the cuts which occur in the axioms of the proof. The clauses are formed depending on how these atoms are related via binary inferences in the proof.

### Definition 20

(*Characteristic clause-set*) Let $\varphi$ be a proof of a skolemized sequent. The characteristic clause set is built recursively from the leaves of the proof to the end-sequent. Let $\nu$ be the occurrence of a sequent in this proof. Then:

- If $\nu$ is an axiom, then $\mathrm{CL}(\nu )$ contains the sub-sequent of $\nu$ composed only of cut-ancestors.
- If $\nu$ is the result of the application of a unary rule on a sequent $\mu$, then $\mathrm{CL}(\nu ) = \mathrm{CL}(\mu )$
- If $\nu$ is the result of the application of a binary rule on sequents $\mu _1$ and $\mu _2$, then we distinguish two cases:
  - If the rule is applied to ancestors of the cut formula, then $\mathrm{CL}(\nu ) = \mathrm{CL}(\mu _1) \cup \mathrm{CL}(\mu _2)$
  - If the rule is applied to ancestors of the end-sequent, then $\mathrm{CL}(\nu ) = \mathrm{CL}(\mu _1) \times \mathrm{CL}(\mu _2)$

where

$$\begin{aligned} \mathrm{CL}(\mu _1) \times \mathrm{CL}(\mu _2) = \{ C \circ D \mid C \in \mathrm{CL}(\mu _1), D \in \mathrm{CL}(\mu _2) \}. \end{aligned}$$

Note that $\circ$ represents the merging of sequents.

If $\nu _0$ is the root node $\mathrm{CL}(\nu _0)$ is called the characteristic clause set of $\varphi$.

### Example 6

The characteristic clause set of our proof $\varphi$ from Example 5 is constructed as follows:

We consider the following cut-ancestors (in axioms) in $\varphi _1$

$$\begin{aligned} \{Pz \vdash \}\text {; } \{\vdash Pf^2 z\}\text {; } \{\vdash f^2 z = gz\} \end{aligned}$$

$=_{r_1}$ operates on cut-ancestors, therefore we get

$$\begin{aligned} S_1 = \{\vdash Pf^2 z \} \text { } \cup \text { } \{ \vdash f^2 z = gz \} \end{aligned}$$

$\rightarrow _l$ operates on end-sequent ancestors, hence

$$\begin{aligned} S = \{Pz \vdash \} \times S_1 = \{Pz \vdash Pf^2 z \text {; } Pz \vdash f^2 z = gz \} \end{aligned}$$

We proceed analogously for the cut-ancestors in $\varphi _2$ and obtain

$$\begin{aligned} S' = \{\vdash Pc \text {; } Pgc \vdash Pgc \text {; } Pg^2 c \vdash \} \end{aligned}$$

The characteristic clause set is $S \cup S'$

$$\begin{aligned} CL(\varphi ) = \{ Pz \vdash Pf^2 z \text {; }\ Pz \vdash f^2 z = gz \text {; }\ \vdash Pc \text {; }\ Pgc \vdash Pgc \text {; }\ Pg^2 c \vdash \}. \end{aligned}$$

The next step is to obtain a resolution refutation of $CL(\varphi )$. It is thus important to show that this set is always refutable.

### Theorem 1

Let $\varphi$ be a proof of a skolemized end-sequent. Then the characteristic clause set $\mathrm{CL}(\varphi )$ is refutable by resolution and paramodulation.

### Proof

In [2, 6]. $$\square $$

### Example 7

We give a PR-refutation $\gamma$ of $\mathrm{CL}(\varphi )$ for $\varphi$ in Example 5:
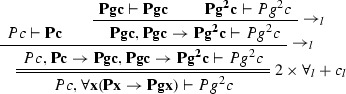
 where $$\pi $$ is
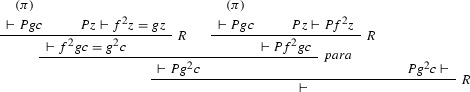


### Definition 21

For a characteristic clause set $\mathrm{CL}(\varphi )$ and a ground refutation R of of a PR refutation $\rho$ of $\mathrm{CL}(\varphi )$ we define

$$\begin{aligned} \mathrm{CL}(\varphi , R) = \{C_i \, | \, C_i \in \mathrm{CL}(\varphi ) \text{ and } C_i \text{ occurs } \text{ in } \text{ R } \}. \end{aligned}$$

Each clause in the clause set will have a projection associated with it. A projection of a clause *C* is a derivation built from $\varphi$ by taking the axioms in which the atoms of *C* occur and all the inferences that operate on end-sequent ancestors. As a result, the end-sequent of a projection will be the end-sequent of $\varphi$ extended by the atoms of *C*.

### Definition 22

(*Projections*) Let $\varphi$ be a proof of a skolemized end-sequent $\varGamma \vdash \varDelta$ in $\mathbf {LK}_=$. For nodes $\nu$ in $\varphi$ we define inductively the set of cut-free proofs $p(\nu )$. If $\nu _0$ is the root node and $\psi \in p(\nu _0)$ we call $\psi$ a *projection*. Let $\nu$ be a node in $\varphi$ such that $S(\nu ) = \varGamma \vdash \varDelta$; then $\varGamma \vdash \varDelta = \varGamma _c,\varGamma _e \vdash \varDelta _e,\varDelta _c$ where $\varGamma _c \vdash \varDelta _c$ consists of cut-ancestors and $\varGamma _e \vdash \varDelta _e$ of ancestors of the end-sequent.

- $\nu$ is a leaf in $\varphi$. Then the sequent at $\nu$ is an axiom and we define $p(\nu ) = \{\nu \}$. The clause part of $\nu$ is the subsequent $\mathrm{CL}(\nu )$.
- $\nu$ is the conclusion of a unary rule $\xi$ with premise $\mu$.
  1. The principal formula of $\xi$ is an ancestor of a cut. Then $\varphi .\nu$ is of the form  We define $p(\nu ) = p(\mu )$.
  2. The principal formula of $\xi$ is an ancestor of the end-sequent. Then $\varphi .\nu$ is of the form  Let $\psi \in p(\mu )$ be a proof of $C,\varGamma _e \vdash \varDelta _e,D$ where $C \vdash D$ is the clause part of $\psi$. Then $\psi ' \in p(\nu )$ for $\psi '=$ and $C \vdash D$ is the clause part of $\psi '$.
- $\nu$ is the conclusion of a binary rule $\xi$ with premises $\mu _1,\mu _2$.
  1. The auxiliary formulas of $\xi$ are ancestors of a cut. Then $\varphi .\nu$ is of the form  Let $\psi \in p(\mu _1)$ such that $\psi$ is a proof of $C,\varGamma _e \vdash \varDelta _e,D$ where $C \vdash D$ is the clause part of $\psi$. Then $\psi ' \in p(\nu )$ for $\psi '=$ and $C \vdash D$ is the clause part of $\psi '$.
    Let $\psi \in p(\mu _2)$ such that $\psi$ is a proof of $E,\varPi _e \vdash \varLambda _e,F$ where $E \vdash F$ is the clause part of $\psi$. Then $\psi ' \in p(\nu )$ for $\psi '=$ and $E \vdash F$ is the clause part of $\psi '$.
  2. The auxiliary formulas of $\xi$ are ancestors of the end-sequent. Then $\varphi .\nu$ is of the form  Let $\psi _1 \in p(\mu _1)$ such that $\psi _1$ is a proof of $C,\varGamma _e \vdash \varDelta _e,D$ and $C \vdash D$ is the clause part of $\psi _1$; likewise let $\psi _2 \in p(\mu _2)$ such that $\psi _2$ is a proof of $E,\varPi _e \vdash \varLambda _e,F$ and $E \vdash F$ is the clause part of $\psi _2$. Then $\psi \in p(\nu )$ for $\psi =$ and the clause part of $\psi$ is $C,E \vdash D,F$.

### Example 8

We define the projections of $\varphi$ from Example 5 to the clauses $Pz \vdash Pf^2 z$, $Pz \vdash f^2 z = gz$, $\vdash Pc$ and $Pg^2c \vdash$. $\varphi [Pz \vdash Pf^2 z]$: 
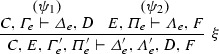
$$\varphi [Pz \vdash f^2 z = gz]$$: 
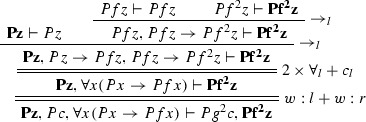
$$\varphi [\vdash Pc]$$: 
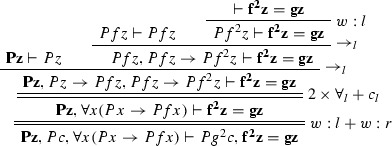


Given the projections and a grounded PR refutation, it is possible to build a proof $\hat{\varphi }$ of $\varGamma \vdash \varDelta$ with only atomic cuts.

If we apply all most general unifiers in the PR proof $\gamma$ we obtain a proof in $\mathbf {LK}_=$ (in fact only contractions, cut and paramodulation remain). If $\gamma \sigma$ is such a proof and we apply a substitution replacing all variables by a constant symbol we obtain a ground PR refutation. Note that after applying the most general unifiers to $\gamma$ we obtain a derivation in $\mathbf {LK}_=$ where the resolution rule becomes a cut rule. For a formal definition see [6].

### Example 9

The ground PR-refutation $\gamma '$ is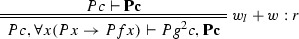 where $$\pi $$ is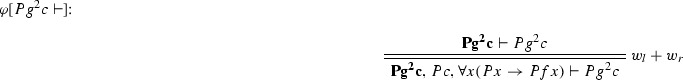 Note that $\gamma '$ is an $\mathbf {LK}_=$-refutation of ground instances of clauses in $\mathrm{CL}(\varphi )$.

$\gamma '$ can be used as a skeleton of a proof $\varphi ^*$ with only atomic cuts of the original end-sequent *S*. $\varphi ^*$ is called a CERES*-normal form* of the original proof $\varphi$. Below we give a formal definition. First we define a type of top normal form defined by a PR-deduction.

### Definition 23

(*Top normal form*) Let $\mathcal{{C}}:\{C_1 \vdash D_1,\ldots ,C_n \vdash D_n\}$ be a set of clauses, $\varGamma \vdash \varDelta$ be a skolemized sequent and $\varphi _i$ cut-free proofs of $C_i,\varGamma \vdash \varDelta ,D_i$ in $\mathbf {LK}_=$ for $i=1,\ldots ,n$. Let $\Phi = \{\varphi _1,\ldots ,\varphi _n\}$. Given a PR-deduction $\varrho$ of a clause $C \vdash D$ from $\mathcal{{C}}$ we define an $\mathbf {LK}_=$-proof $\varTheta (\varrho ,\mathcal{{C}},\Phi )$ of $C,\varGamma \vdash \varDelta ,D$ inductively on the length of $\varrho$.

- $$\varrho = C_i \vdash D_i$$: then $$\varTheta (\varrho ,\mathcal{{C}},\Phi ) = \varphi _i$$ and $${ top}(\varTheta (\varrho ,\mathcal{{C}},\Phi )) = \{\varphi _i\}$$.
- The last inference in $\varrho$ is *R*. Then $\varrho$ is of the form  Let us assume that  Then we define $\varTheta (\varrho ,\mathcal{{C}},\Phi ) =$ and ${ top}(\varTheta (\varrho ,\mathcal{{C}},\Phi )) = { top}(\varTheta (\varrho _1,\mathcal{{C}},\Phi )) \cup { top}(\varTheta (\varrho _1,\mathcal{{C}},\Phi ))$.
- The last inference in $\varrho$ is a paramodulation rule. We consider only the case $=_{r1}$; for the other rules the construction is analogous. Then $\varrho$ is of the form  Let us assume that  Then we define $\varTheta (\varrho ,\mathcal{{C}},\Phi ) =$ and ${ top}(\varTheta (\varrho ,\mathcal{{C}},\Phi )) = { top}(\varTheta (\varrho _1,\mathcal{{C}},\Phi )) \cup { top}(\varTheta (\varrho _1,\mathcal{{C}},\Phi ))$.

A proof $\psi$ is called in *top normal form* if there are $\mathcal{{C}},\Phi$ and $\rho$ (defined as above) such that $\psi = \varTheta (\rho ,\mathcal{{C}},\Phi )$.

### Remark 2

The function ${ top}$ collects all cut-free subproofs in a top normal form which occur at the top and thus belong to $\Phi$.

### Definition 24

(CERES-*normal form*) Let $\varphi$ be an $\mathbf {LK}_=$ proof of a skolemized sequent *S*. Let $\varrho$ be a grounded PR-refutation of $\mathrm{CL}(\varphi )$, $\mathcal{{C}}$ be the set of all ground instances of clauses in $\mathrm{CL}(\varphi )$ appearing at the leaves of $\varrho$ and $\Phi$ be the set of all grounded projections. Then the proof $\varTheta (\varrho ,\mathcal{{C}},\Phi )$ is called a CERES* normal form* of $\varrho$. As $\varrho$ is a refutation $\varTheta (\varrho ,\mathcal{{C}},\Phi )$ is a proof of *S* with only atomic cuts.

### Remark 3

Note that not all top normal forms are CERES normal forms as the set of cut-free proofs $\Phi$ need not be projections.

### Example 10

We define a CERES normal form for the proof from Example 5 with respect to the grounded resolution refutation $\gamma '$ of $\mathrm{CL}(\varphi )$ (in the following example $F = \forall x(Px \rightarrow Pfx)$):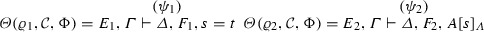 where $$\varphi _1$$ is
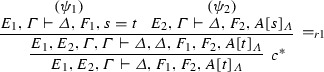
$$\varphi _{11}$$ is
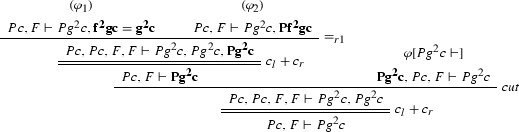
$$\varphi _2$$ is
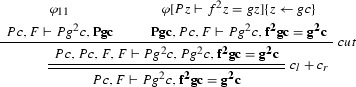
$$\pi _1$$ is
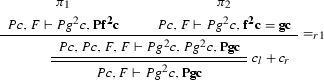
 and $$\pi _2$$ is
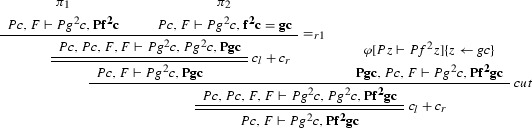


## Extraction of Expansion Trees from Projections

The extraction of expansion proofs is usually performed after the construction of a proof in top normal form. However, only the *logical* parts of the proof play a role in the construction of expansion trees. These logical parts can be identified as the cut-free subproofs after removal of all cut-ancestors. Note that no cut-ancestor in such a subproof is principal formula of an inference; we identify such subsequents as *passive subsequent*.

### Definition 25

(*Passive subsequent*) Let $\varphi$ be a cut-free proof of $S: C, \varGamma \vdash \varDelta , D$ such that $C \vdash D$ is a clause. The subsequent $C \vdash D$ of *S* is called *passive* in $\varphi$ if no ancestor of $C \vdash D$ in $\varphi$ contains a formula which is principal formula of an inference.

Note that the passive subsequents are just the clauses used to define a top normal form. Examples of proofs with passive clause parts are proof projections in CERES:

### Proposition 2

Let $\psi$ be a cut-free proof of $C',\varGamma \vdash \varDelta ,D'$ which is an instance of a proof projection $\varphi [C \vdash D]$ in CERES. Then $C' \vdash D'$ is passive in $\psi$.

### Proof

Immediate by induction on the length of $\psi$ and by Definition 22. Note that the only case in Definition 22 where the clause part changes is (c2). Here the projection $\psi$ is defined asBy induction hypothesis $C \vdash D$ is passive in $\psi _1$ and $E \vdash F$ is passive in $\psi _2$. Therefore $C,E \vdash D,F$ is passive in $\psi$. $\square$

### Definition 26

Let $\varphi$ be a cut-free proof of $C, \varGamma \vdash \varDelta , D$ where $C \vdash D$ is passive in $\varphi$. We define $\varphi \backslash (C \vdash D)$ by induction on the number of nodes in $\varphi$.

- If $\varphi$ is an axiom then $\varphi = C, C' \vdash D, D'$ (note that the whole sequent is passive in $\varphi$). We define $\varphi \backslash (C \vdash D) = C' \vdash D'$.
- Let $$\varphi = $$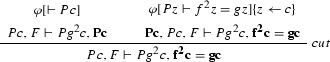 where $C \vdash D$ is passive in $\varphi$. Then, by definition of passive subclauses, $\varphi '$ is a proof of $C, \varGamma ' \vdash \varDelta ', D$ for some $\varGamma '$ and $\varDelta '$. Indeed, the subclause $C \vdash D$ does not contain a formula which is principal formula of an inference. By induction we have a proof $\varphi ' \backslash (C \vdash D)$ of $\varGamma ' \vdash \varDelta '$ (note that $C \vdash D$ is also passive in $\varphi '$) and we define $\varphi \backslash (C \vdash D) =$
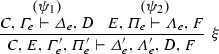
- Let $$\varphi =$$
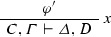
 where $C \vdash D$ is passive in $\varphi$. As *C*, *D* are not principal formulas of an inference we get that $S_1 = C_1 , \varGamma _1 \vdash \varDelta _1 , D_1$, $S_2 = C_2 , \varGamma _2 \vdash \varDelta _2 , D_2$, s.t. $C_1 , C_2 \vdash D_1, D_2 = C \vdash D$ and $C_1 \vdash D_1$ is passive in $\varphi _1$, $C_2 \vdash D_2$ is passive in $\varphi _2$.
  By induction we have a proof $\varphi _1 \backslash (C_1 \vdash D_1 )$ of $\varGamma _1 \vdash \varDelta _1$ and a proof $\varphi _2 \backslash (C_2 \vdash D_2)$ of $\varGamma _2 \vdash \varDelta _2$. Then we obtain $\varphi \backslash (C \vdash D) =$
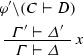

The function $\mathrm{logical}(\varphi )$ for a proof in top normal form takes the cut-free proofs on top and “subtracts” from them all ancestors of passive clauses.

### Definition 27

(logical$$(\varphi )$$) Let $$\varphi :\varTheta (\rho ,\mathcal{{C}},\Phi )$$ be a proof in top normal form s.t. $$\mathcal{{C}}= \{C_1 \vdash D_1,\ldots , C_n \vdash D_n\}$$ and $$\Phi = \{\varphi _1,\ldots ,\varphi _n\}$$ such that $$\varphi _i$$ is a cut-free proof of $$C_i,\varGamma \vdash \varDelta ,D_i$$. Assume that for all $$i=1,\ldots ,n$$$$C_i \vdash D_i$$ is passive in $$\varphi _i$$. For every $$\psi \in { top}(\varphi )$$ and $$\psi = \varphi _i$$ we define $$\psi ' = \varphi _i \backslash (C_i \vdash D_i)$$ and $$\mathrm{logical}(\varphi ) = \{\psi ' \mid \psi \in { top}(\varphi )\}$$.

Below we define an expansion tree $\hat{E}(\varphi )$ which is defined by merging the expansion trees of $\mathrm{logical}(\varphi )$. This structure will be the key for the development of an efficient algorithm for extracting expansion trees from CERES normal forms.

### Definition 28

Let $\varphi$ be a proof in top normal form of a skolemized and normalized end-sequent. We define

$$\begin{aligned} \hat{E}(\varphi ) = {\mathrm{merge}}\{\mathrm{ET}(\psi ) \mid \psi \in \mathrm{logical}(\varphi )\}. \end{aligned}$$

### Theorem 2

Let $$\varphi :\ \varTheta (\varrho ,\mathcal{{C}},\Phi )$$ be a proof of a skolemized and normalized sequent $$C,\varGamma \vdash \varDelta ,D$$ in top normal form such that $$\mathcal{{C}}= \{C_1 \vdash D_1,\ldots , C_n \vdash D_n\}$$ and $$\Phi = \{\varphi _1,\ldots ,\varphi _n\}$$, where $$\varphi _i$$ is a cut-free proof of $$C_i,\varGamma \vdash \varDelta ,D_i$$. Assume that for all $$i=1,\ldots ,n$$$$C_i \vdash D_i$$ is passive in $$\varphi _i$$. Then $$\mathrm{ET}(\varphi ) = \hat{E}(\varphi ) \circ (C \vdash D)$$.

### Proof

By induction on the number of nodes in $\varrho$.

*Case 1:* if $\rho$ consists of just one node then $\varphi = \varphi _i$ for some $i \in \{1,\ldots ,n\}$ . We have to show that $\mathrm{ET}(\varphi _i) = \hat{E}(\varphi _i)\circ (C_i \vdash D_i)$. But $\hat{E}(\varphi _i) = \mathrm{ET}(\varphi _i \backslash (C_i \vdash D_i))$ and thus it remains to show that

$$\begin{aligned} (\star )\ \mathrm{ET}(\varphi _i) = \mathrm{ET}(\varphi _i \backslash (C_i \vdash D_i)) \circ (C_i \vdash D_i). \end{aligned}$$

$(\star )$ is obtained via an easy induction on the number of inferences in $\varphi _i$ using Definition 26.

*Case 2:* The last inference in $\varrho$ is *R*. Then $\varrho$ is of the formThen (by definition of $\varphi$ as $\varTheta (\varrho ,\mathcal{{C}},\Phi )$) $\varphi$=Assume that

$$\begin{aligned} \mathrm{ET}(\varphi _1)= & {} C_1,\varGamma ^* \vdash \varDelta ^*,D_1,A^m,\\ \mathrm{ET}(\varphi _2)= & {} A^k,C_2,\varGamma ^+ \vdash \varDelta ^+,D_2, \end{aligned}$$

where ${ Seq}(\varGamma ^* \vdash \varDelta ^*) = { Seq}(\varGamma ^+ \vdash \varDelta ^+)$. By Definition 19 we obtain

$$\begin{aligned} (1)\ \mathrm{ET}(\varphi ) = (C_1,C_2 \vdash D_1,D_2) \circ \mathrm{merge}(\varGamma ^* \vdash \varDelta ^*, \varGamma ^+ \vdash \varDelta ^+) \end{aligned}$$

Note that $\varGamma ^*,\varGamma ^+$ and $\varDelta ^*,\varDelta ^+$ are normalized. By induction hypothesis we have

$$\begin{aligned} \hat{E}(\varphi _1) \circ (C_1 \vdash D_1,A^m)= & {} \mathrm{ET}(\varphi _1),\\ \hat{E}(\varphi _2) \circ (A^k,C_2 \vdash D_2)= & {} \mathrm{ET}(\varphi _2). \end{aligned}$$

and therefore

$$\begin{aligned} (2)\ \hat{E}(\varphi _1) = \varGamma ^* \vdash \varDelta ^*,\ \ \hat{E}(\varphi _2) = \varGamma ^+ \vdash \varDelta ^+. \end{aligned}$$

By definition of the merge operator we get from (1) and (2)

$$\begin{aligned} (3)\ \mathrm{ET}(\varphi ) = (C_1,C_2 \vdash D_1,D_2) \circ \mathrm{merge}(\hat{E}(\varphi _1),\hat{E}(\varphi _2)). \end{aligned}$$

By Definition 28 we obtain

$$\begin{aligned} \hat{E}(\varphi _1)= & {} \mathrm{merge}\{\mathrm{ET}(\psi ) \mid \psi \in \mathrm{logical}(\varphi _1)\},\\ \hat{E}(\varphi _2)= & {} \mathrm{merge}\{\mathrm{ET}(\psi ) \mid \psi \in \mathrm{logical}(\varphi _2)\}. \end{aligned}$$

Hence

$$\begin{aligned} \begin{array}{l} \mathrm{merge}(\hat{E}(\varphi _1),\hat{E}(\varphi _2)) = \\ \mathrm{merge}(\mathrm{merge}\{\mathrm{ET}(\psi ) \mid \psi \in \mathrm{logical}(\varphi _1)\},\mathrm{merge}\{\mathrm{ET}(\psi ) \mid \psi \in \mathrm{logical}(\varphi _2)\}) = \\ \mathrm{merge}\{\mathrm{ET}(\psi ) \mid \psi \in \mathrm{logical}(\varphi )\}. \end{array} \end{aligned}$$

But by Definition 28$\hat{E}(\varphi ) = \mathrm{merge}\{\mathrm{ET}(\psi ) \mid \psi \in \mathrm{logical}(\varphi )\}$. Combing this with (3) we obtain

$$\begin{aligned} \mathrm{ET}(\varphi ) = (C_1,C_2 \vdash D_1,D_2) \circ \hat{E}(\varphi ) . \end{aligned}$$

*Case 3:* The last inference in $\varrho$ is a paramodulation. We only consider the case $=_{r_1}$, the others are analogous. Then $\varrho$ is of the formThen, by definition of $\varphi$, we obtain $\varphi$=Assume that

$$\begin{aligned} \mathrm{ET}(\varphi _1)= & {} C_1,\varGamma ^* \vdash \varDelta ^*,D_1,s=t,\\ \mathrm{ET}(\varphi _2)= & {} C_2,\varGamma ^+ \vdash \varDelta ^+,D_2,A[s] \end{aligned}$$

where ${ Seq}(\varGamma ^* \vdash \varDelta ^*) = { Seq}(\varGamma ^+ \vdash \varDelta ^+)$. By Definition 19 we obtain

$$\begin{aligned} (4)\ \mathrm{ET}(\varphi ) = (C_1,C_2 \vdash D_1,D_2,A[t]) \circ \mathrm{merge}(\varGamma ^* \vdash \varDelta ^*, \varGamma ^+ \vdash \varDelta ^+). \end{aligned}$$

By induction hypothesis we have

$$\begin{aligned} \hat{E}(\varphi _1) \circ (C_1 \vdash D_1,s=t)= & {} \mathrm{ET}(\varphi _1),\\ \hat{E}(\varphi _2) \circ (C_2 \vdash D_2,A[s])= & {} \mathrm{ET}(\varphi _2). \end{aligned}$$

and therefore

$$\begin{aligned} (5)\ \hat{E}(\varphi _1) = \varGamma ^* \vdash \varDelta ^*,\ \ \hat{E}(\varphi _2) = \varGamma ^+ \vdash \varDelta ^+. \end{aligned}$$

By definition of the merge operator we get from (4) and (5)

$$\begin{aligned} (6)\ \mathrm{ET}(\varphi ) = (C_1,C_2 \vdash D_1,D_2,A[t]) \circ \mathrm{merge}(\hat{E}(\varphi _1),\hat{E}(\varphi _2)). \end{aligned}$$

By Definition 28 we obtain

$$\begin{aligned} \hat{E}(\varphi _1)= & {} \mathrm{merge}\{\mathrm{ET}(\psi ) \mid \psi \in \mathrm{logical}(\varphi _1)\},\\ \hat{E}(\varphi _2)= & {} \mathrm{merge}\{\mathrm{ET}(\psi ) \mid \psi \in \mathrm{logical}(\varphi _2)\}. \end{aligned}$$

Hence, like in Case 2, we get

$$\begin{aligned} \mathrm{merge}(\hat{E}(\varphi _1),\hat{E}(\varphi _2)) = \mathrm{merge}\{\mathrm{ET}(\psi ) \mid \psi \in \mathrm{logical}(\varphi )\}. \end{aligned}$$

But by Definition 28$\hat{E}(\varphi ) = \mathrm{merge}\{\mathrm{ET}(\psi ) \mid \psi \in \mathrm{logical}(\varphi )\}$. Combing this with (6) we obtain

$$\begin{aligned} \mathrm{ET}(\varphi ) = (C_1,C_2 \vdash D_1,D_2,A[t]) \circ \hat{E}(\varphi ) . \end{aligned}$$

$$\square $$

### Corollary 1

Let $$\varphi :\varTheta (\varrho ,\mathcal{{C}},\Phi )$$ be a proof of a skolemized and normalized sequent $$\varGamma \vdash \varDelta $$ in top normal form s.t. $$\mathcal{{C}}= \{C_1 \vdash D_1,\ldots , C_n \vdash D_n\}$$ and $$\Phi = \{\varphi _1,\ldots ,\varphi _n\}$$ such that $$\varphi _i$$ is a cut-free proof of $$C_i,\varGamma \vdash \varDelta ,D_i$$. Assume that for all $$i=1,\ldots ,n$$$$C_i \vdash D_i$$ is passive in $$\varphi _i$$. Then $$\mathrm{ET}(\varphi ) = \hat{E}(\varphi )$$.

### Proof

Immediate by Theorem 2: just define $C \vdash D$ as the empty sequent. $\square$

### Corollary 2

Let $\varphi$ be an $\mathbf {LK}_=$ proof of a skolemized and normalized sequent *S*. Let $\varphi ^*:\varTheta (\varrho ,\mathcal{{C}},\Phi )$ be a CERES normal form of $\varphi$ such that $\varrho$ is a ground PR-refutation of $\mathcal{{C}}$, the set of all ground instances of clauses in $\mathrm{CL}(\varphi )$, and $\Phi$ is the set of all grounded projections. Then $\mathrm{ET}(\varphi ) = \hat{E}(\varphi )$.

### Proof

Let $\psi$ be a cut-free proof of $C,\varGamma \vdash \varDelta ,D$ which is an instance of a projection of $\varphi$. By Proposition 2$C \vdash D$ is passive in $\psi$. As CERES normal forms are top normal forms all conditions of Corollary 1 are fulfilled. $\square$

The last corollary describes a method to compute an expansion tree from any proof in top normal form of a skolemized sequent *S*. Note that in case of a prenex sequent *S* we extract Herbrand sequents. The computation of an expansion tree is based on top normal forms. CERES normal forms $\varphi ^{*}$ of proofs $\varphi$ are also in top normal form, therefore we can compute expansion trees in the same way. For CERES it means that $\varphi ^{*}$ has to be constructed first. The usual algorithm for the extraction of expansion trees from the CERES normal form is:

$$\begin{aligned} \begin{array}{l} \mathbf {begin}\ \ \% \ \text{ algorithm } \text{ EXP }\\ 1. \text{ compute } \mathrm{CL}(\varphi );\\ 2. \text{ find } \text{ a } \text{ PR } \text{ refutation } \rho \text{ of } \mathrm{CL}(\varphi );\\ 3. \text{ compute } \text{ a } \text{ ground } \text{ refutation } R \text{ from } \rho ;\\ 4. \text{ compute } \text{ the } \text{ projections } \varphi [C] \text{ for } C \in \mathrm{CL}(\varphi , R);\\ 5. \text{ construct } \text{ the } \text{ CERES } \text{ normal } \text{ form } \varphi ^* \text{ from } \text{ the } \text{ projections } \text{ and } R;\\ 6. \text{ extract } \text{ an } \text{ expansion } \text{ proof } \mathrm{ET}(\varphi ) \text{ from } \varphi ^* . \\ \mathbf {end}. \end{array} \end{aligned}$$

Instead of using algorithm EXP, we make use of Theorem 2 and define a new method that extracts expansion proofs more efficiently by extracting partial expansion trees from the projections. The idea is the following: we do not compute $\mathrm{logical}(\varphi ^{*})$ which would be the set of all instantiated projections. Note that the size of $\mathrm{logical}(\varphi ^{*})$ is roughly the size of $\varphi ^{*}$ itself. Instead we use from a ground resolution refutation *R* of $\mathrm{CL}(\varphi )$ the general projections $\varphi [C]$ for $C \in \mathrm{CL}(\varphi , R)$ and the set of substitutions $\varSigma (C)$ for $C \in \mathrm{CL}(\varphi , R)$ which are the set of ground substitutions for the clause *C* in the refutation *R*.

### Definition 29

For every projection $\varphi [C]:\mathbf {A}, \varGamma \vdash \varDelta , \mathbf {B}$, where $\mathbf {A} \vdash \mathbf {B}$ is the clause part of $\varphi [C]$, we define $\varphi ^{-} [C] = \varphi (C) \backslash (\mathbf {A} \vdash \mathbf {B})$ (note that $\mathbf {A} \vdash \mathbf {B}$ is a passive subsequent of $\mathbf {A}, \varGamma \vdash \varDelta , \mathbf {B}$), where the $\backslash$-operator is defined as in Definition 26. Note that $\mathrm{ET}(\varphi ^{-} [C])$ is a proof relative to the axioms in $\varphi ^{-} [C]$, which may differ from the axioms of $\varphi$ (axioms need not be tautological anyway). We define

$$\begin{aligned} T(\varphi , R) = {\mathrm{merge}}_{C \in \mathrm{CL}(\varphi , R)} {\mathrm{merge}}_{\sigma \in \varSigma (C)} \mathrm{ET}(\varphi ^{-} [C])\sigma . \end{aligned}$$

Then the computation of an expansion tree via CERES for a proof $\varphi$ (of a closed skolemized end-sequent *S*) goes as follows:

$$\begin{aligned} \begin{array}{l} \mathbf {begin}\ \ \% \ \text{ algorithm } \text{ EXP }_{new}\\ 1. \text{ compute } \mathrm{CL}(\varphi );\\ 2. \text{ find } \text{ a } \text{ PR } \text{ refutation } \rho \text{ of } \mathrm{CL}(\varphi );\\ 3. \text{ compute } \text{ a } \text{ ground } \text{ refutation } R \text{ from } \rho \\ \quad \text{ and } \text{ for } \text{ every } C \in \mathrm{CL}(\varphi , R) \text{ the } \text{ set } \varSigma (C);\\ 4. \text{ compute } \text{ the } \text{ projections } \varphi [C] \text{ and } \varphi ^-[C] \text{ for } C \in \mathrm{CL}(\varphi , R);\\ 5. \text{ for } \text{ every } C \in \mathrm{CL}(\varphi , R) \text{ compute } T[C]:{\mathrm{merge}}_{\sigma \in \varSigma (C)}\mathrm{ET}(\varphi ^-[C])\sigma ;\\ 6. \text{ compute } {\mathrm{merge}}_{C \in \mathrm{CL}(\varphi , R)}T[C] \text{ which } \text{ is } T(\varphi ,R). \\ \mathbf {end}. \end{array} \end{aligned}$$

Note that the computation of the ${ Dp}$ function of an expansion proof via CERES for a proof $\varphi$ (of a closed skolemized end-sequent *S*) can be easily obtained by computing the ${ Dp}$ function of $T(\varphi , R)$.

### Theorem 3

Let $\varphi$ be a proof of a skolemized, closed and normalized end-sequent and $\varphi ^{*}$ the CERES normal form based on a ground refutation *R* of $\mathrm{CL}(\varphi )$. Then $\mathrm{ET}(\varphi ^{*}) = T(\varphi , R)$.

### Proof

Let $\mathrm{CL}(\varphi , R) = \{C_1,\ldots ,C_n\}$. Now ${\mathrm{logical}}(\varphi ^{*}) = \Phi _{1} \cup \cdots \cup \Phi _{n}$ where $\Phi _{i} = \{ \varphi ^{-}[C_{i}] \sigma \, | \, \sigma \in \varSigma (C_i) \}$. Let $\psi _i = \varphi ^{-}[C_{i}]$. Then by Theorem 2 we know that

$$\begin{aligned} \mathrm{ET}(\varphi ^{*}) = {\mathrm{merge}}( {\mathrm{merge}}_{\sigma \in \varSigma (C_1)} \mathrm{ET}(\psi _1\sigma ), \cdots , {\mathrm{merge}}_{\sigma \in \varSigma (C_n)} \mathrm{ET}(\psi _n\sigma )) \end{aligned}$$

which is equal to

$$\begin{aligned} \mathrm{ET}(\varphi ^{*}) = {\mathrm{merge}}( {\mathrm{merge}}_{\sigma \in \varSigma (C_1)} \mathrm{ET}(\psi _1)\sigma , \cdots , {\mathrm{merge}}_{\sigma \in \varSigma (C_n)} \mathrm{ET}(\psi _n)\sigma ) \end{aligned}$$

which, by Definition 29, is exactly $T(\varphi ,R)$. So $\mathrm{ET}(\varphi ^*) =T(\varphi ,R)$. $\square$

Note that Theorem 3 also holds for the ${ Dp}$ function of expansion proofs, i.e. ${ Dp}(\mathrm{ET}(\varphi ^{*})) = { Dp}(T(\varphi ,R))$.

### Corollary 3

Let $\varphi$ be a proof of a skolemized,closed and normalized sequent *S* and *R* be a refutation of $\mathrm{CL}(\varphi )$. Then $T(\varphi ,R)$ is an expansion proof of *S*.

### Proof

By Theorems 2 and 3. $\square$

Instead of computing all $\varphi [C_{j}] \sigma ^{j}_{i}$ (obtained from the ACNF $\varphi ^*$) the algorithm EXP$_{new}$ computes the $\varphi [C_{j}]$ and extracts $\mathrm{ET}(\varphi ^{-}[C_{j}]) \equiv T_j$, which is a partial expansion proof, then constructs $\times _{\sigma \in \varSigma (C_j)}T_j\sigma$ for all *j* and merges them. Example 11 illustrates the main features of the method.

### Example 11

Consider the proof $\varphi$ as in Example 5 (where $F = \forall x(Px \rightarrow Pfx)$). The ACNF $\varphi$ is:
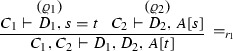
 where $$\varphi _1$$ is
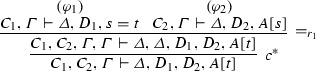
$$\varphi _{1_1}$$ is
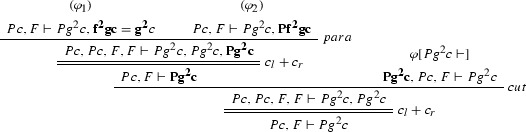
$$\varphi _2$$ is
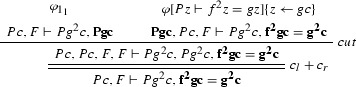
$$\pi _1$$ is
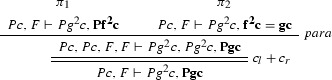
 and $$\pi _2$$ is
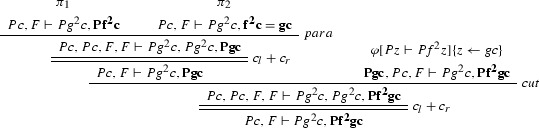
 Now compute the Herbrand sequent of $\varphi ^*$ (with the old method):

$$\begin{aligned} \begin{array}{lcl} H(\varphi ^{*})= & {} Pc, Pc \rightarrow Pfc, Pfc \rightarrow Pf^2 c, Pgc \rightarrow Pfgc, Pfgc \rightarrow Pf^2 gc \vdash Pg^2 c \end{array} \end{aligned}$$

Note that $H(\varphi ^{*})$ is a valid sequent in our axiom set ($\vdash f^2 z = gz$ is an axiom).

With our new method we first compute $T_{i} = \mathrm{ET}(\varphi ^{-} [C_{i}])$ and define the substitutions $\sigma _{i}^{j}$:

$$\begin{aligned} \begin{array}{lcl} T_{1} &{} = &{} Pc, \forall x (Px \rightarrow Pfx) +^{z} Pz \rightarrow Pfz +^{fz} Pfz \rightarrow Pf^2 z \vdash Pg^2 c \\ T_{2} &{} = &{} Pc, \forall x (Px \rightarrow Pfx) +^{z} Pz \rightarrow Pfz +^{fz} Pfz \rightarrow Pf^2 \vdash Pg^2 c \\ T_{3} &{} = &{} Pc, \forall x (Px \rightarrow Pfx) \vdash Pg^2 c \\ T_{4} &{} = &{} Pc, \forall x (Px \rightarrow Pfx) \vdash Pg^2 c \\ \sigma ^{1}_{1} &{} = &{} (z \leftarrow c) \quad \sigma ^{1}_{2} = (z \leftarrow gc) \\ \sigma ^{2}_{1} &{} = &{} (z \leftarrow c) \quad \sigma ^{2}_{2} = (z \leftarrow gc) \\ \end{array} \end{aligned}$$

Note that $$T_1 = T_2$$. Now we compute $$T(\varphi , R) =$$$${\mathrm{merge}}(T_{1}$$$$\sigma ^{1}_{1},$$$$T_{1}$$$$\sigma ^{1}_{2},$$$$T_{2}$$$$\sigma ^{2}_{1},$$$$T_{2}$$$$\sigma ^{2}_{2}, T_{3}, T_{4})$$

$$\begin{aligned}&\begin{array}{lcl} T(\varphi , R) &{} = &{} {\mathrm{merge}}(Pc, \forall x (Px \rightarrow Pfx) +^{c} Pc \rightarrow Pfc +^{fc} Pfc \rightarrow Pf^2 c \\ &{} &{} \vdash Pg^2 c, \\ &{} &{} (Pc, \forall x (Px \rightarrow Pfx) +^{gc} Pgc \rightarrow Pfgc +^{fgc} Pfgc \rightarrow Pf^2 gc\\ &{} &{} \vdash Pg^2 c, \\ &{} &{} Pc, \forall x (Px \rightarrow Pfx) \vdash Pg^2 c, \\ &{} &{} Pc, \forall x (Px \rightarrow Pfx) \vdash Pg^2 c) \end{array} \\&\begin{array}{ccclc} T(\varphi , R) &{} = &{} Pc, \forall x (Px \rightarrow Pfx) &{} +^{c} Pc \rightarrow Pfc,&{} \\ &{} &{} &{} +^{fc} Pfc \rightarrow Pf^2 c, &{} \\ &{} &{} &{} +^{gc} Pgc \rightarrow Pfgc, &{} \\ &{} &{} &{} +^{fgc} Pfgc \rightarrow Pf^2 gc &{} \vdash Pg^2 c \end{array} \end{aligned}$$

The ${ Dp}$ function is

$$\begin{aligned} { Dp}(T(\varphi , R) )) = Pc, Pc \rightarrow Pfc, Pfc \rightarrow Pf^2 c, Pgc \rightarrow Pfgc, Pfgc \rightarrow Pf^2 gc \vdash Pg^2 c \end{aligned}$$

## Complexity

In this section we prove that the algorithm $\mathrm{EXP}_{new}$ outperforms the old algorithm $\mathrm{EXP}$. In particular we prove that the complexity of $\mathrm{EXP}_{new}$ is always better or equal to that of $\mathrm{EXP}$. Then we define an infinite sequence of **LK**-proofs $\varphi _n$ where the complexity of $\mathrm{EXP}$ is cubic in *n* while that of $\mathrm{EXP}_{new}$ is only quadratic. This implies that the computational complexity of $\mathrm{EXP}$ cannot be linearly bounded by that of $\mathrm{EXP}_{new}$. Our complexity measure will be the maximal logical complexity of objects constructed by the algorithms.

### Definition 30

(*Size of a formula*) Let *A* be a formula, then the size of *A* ($\Vert A \Vert _f$) is inductively defined as follows

$$\begin{aligned} \begin{array}{lcl} \Vert A \Vert _f &{} = &{} 1 \text { if } A \text { is an atomic formula }, \\ \Vert \lnot A \Vert _f &{} = &{} 1 + \Vert A \Vert _f, \\ \Vert A_1 \circ A_2 \Vert _f &{} = &{} 1 + \Vert A_1 \Vert _f + \Vert A_2 \Vert _f, \circ \in \{\wedge , \vee , \rightarrow \}, \\ \Vert Qx.A \Vert _f &{} = &{} 1 + \Vert A \Vert _f, Q \in \{\forall , \exists \}. \end{array} \end{aligned}$$

To improve legibility we write $\Vert F \Vert$ instead of $\Vert F \Vert _f$ (with the exception of cases where the precise notation is essential) and use the measure $\Vert \Vert$ also for sequents, proofs and clause sets.

### Definition 31

(*Size of a sequent*) Let $S: A_1 , \ldots , A_n \vdash A_{n+1} , \ldots , A_m$ be a sequent, then the size of *S* is

$$\begin{aligned} \Vert A_1 , \ldots , A_n \vdash A_{n+1} , \ldots , A_m \Vert = \varSigma ^{m}_{i = 1} \Vert A_i \Vert \end{aligned}$$

### Definition 32

(*Size of an*$\mathbf {LK}_=$-*proof*) Let $\varphi$ be an $\mathbf {LK}_=$-proof. If $\varphi$ is an axiom then $\varphi$ consists of just one node labelled by a sequent *S*; here we define $\Vert \varphi \Vert = \Vert S \Vert$.

If $\varphi$ is not an axiom then the end-sequent is a conclusion of a unary or of a binary rule. So we distinguish two cases:

(a) $$\varphi =$$Then $$\Vert \varphi \Vert = \Vert \varphi ' \Vert + \Vert S \Vert $$.

(b) $$\varphi =$$Then $$\Vert \varphi \Vert = \Vert \varphi _1 \Vert + \Vert \varphi _2 \Vert + \Vert S \Vert $$.

### Definition 33

(*Size of an expansion tree*) Let *E* be an expansion tree, then the size of *E* ($\Vert E \Vert$) is inductively defined as follows

$$\begin{aligned} \begin{array}{lcl} \Vert E \Vert &{} = &{} \Vert E \Vert _f \text { if } E \text { is a quantifier-free formula }, \\ \Vert \lnot E \Vert &{} = &{} 1 + \Vert E \Vert , \\ \Vert E_1 \circ E_2 \Vert &{} = &{} 1 + \Vert E_1 \Vert + \Vert E_2 \Vert , \circ \in \{\wedge , \vee , \rightarrow \}, \\ \Vert Qx.E +^{t_1} E_{1} +^{t_2} \cdots +^{t_n} E_{n} \Vert &{} = &{} 1 + \Vert E \Vert + \Vert E_{1} \Vert + \cdots + \Vert E_{n} \Vert , Q \in \{\forall , \exists \}. \end{array} \end{aligned}$$

### Definition 34

(*Size of an s-expansion proof*) Let $E: E_1 , \ldots , E_n \vdash E_{n+1}, \ldots , E_m$ be an s-expansion proof, then the size of *E* ($\Vert E \Vert$) is

$$\begin{aligned} \Vert E_1 , \ldots , E_n \vdash E_{n+1}, \ldots , E_m \Vert = \varSigma ^{m}_{i = 1} \Vert E_i \Vert . \end{aligned}$$

In our algorithms $\mathrm{EXP}$ and $\mathrm{EXP}_{new}$ we do not only construct sequents, formulas and proofs, but also sets of clauses (which are finite sets of atomic sequents). If $\mathcal{{C}}= \{C_1,\ldots ,C_n\}$ we define $\Vert \mathcal{{C}} \Vert = \Vert C_1 \Vert + \ldots + \Vert C_n \Vert$. We call the objects produced by a proof transformation expressions.

### Definition 35

(*Expression*) An *expression* is a formula, a sequent, a proof or a set of clauses.

Now we consider computations as sequences of expressions which are generated by an algorithmic proof transformation. So let *A* be an algorithm and $\varphi$ be a proof serving as input to *A*. Then $E_A(\varphi )$ is the sequence of all expressions generated by *A* on input $\varphi$. Below we define a complexity function induced by *A* given by the maximal expression generated by *A*:

### Definition 36

Let *A* be an algorithm on proofs. Then we define

$$\begin{aligned} C_A(\varphi ) = \max \{\Vert x \Vert \mid x \in E_A(\varphi )\}. \end{aligned}$$

### Theorem 4

$C_{\mathrm{EXP}_{new}}(\varphi ) \le C_{\mathrm{EXP}}(\varphi )$ for all proofs $\varphi$ in $\mathbf {LK}_=$.

### Proof

sketch: the first 4 steps of $\mathrm{EXP}$ and $\mathrm{EXP}_{new}$ are identical. The sum of the sizes of the expansion trees generated by $\mathrm{EXP}_{new}$ is smaller or equal to the size of the CERES normal form generated by $\mathrm{EXP}$. $\square$

We show now that $\mathrm{EXP}_{new}$ can be asymptotically better that $\mathrm{EXP}$. To this aim we define the following sequence of **LK**-proofs $\varphi _n$: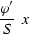 where $$\omega _n$$ is: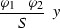 and $$\pi _n$$ is: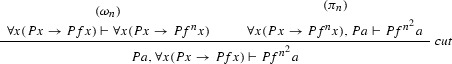 recursive definition of $$\psi _n{:}\,\psi _0 = Py \vdash Py$$. And for $$n>0$$$$\psi _n=$$
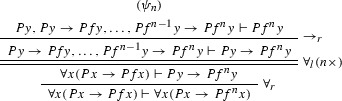
 For our complexity measure we obtain

$$\begin{aligned} \Vert \psi _n \Vert = 2 + 2(n+1) + \Vert \psi _{n-1} \Vert \end{aligned}$$

note that $$\Vert \psi _{n-1} \Vert = \Vert \psi _{n-1} \{y \leftarrow fy\} \Vert $$.

$$\begin{aligned} C_P (\psi _0) = 2 \end{aligned}$$

Obviously, there are constants $a_1,a_2,b_1,b_2$ (all $>0$) such that

$$\begin{aligned} a_{1}*n^2 \le \Vert \psi _n \Vert \le a_{2}*(n+1)^2\ \text{ and }\ b_{1}*n^2 \le \Vert \chi _n \Vert \le b_{2}*(n+1)^2 . \end{aligned}$$

Putting things together there are constants $c_1,c_2 >0$ with

$$\begin{aligned} c_1*n^2 \le \Vert \varphi _n \Vert \le c_2*(n+1)^2. \end{aligned}$$

Now we compute the characteristic clause sets of the $\varphi _n$. After elimination of tautologies we get

$$\begin{aligned} CL(\varphi _{n}) = \{ C_{1,n} : Py \vdash Pf^{n}y; \; C_2 : \; \vdash Pa; \; C_{3,n} : Pf^{n^2}a \vdash \}. \end{aligned}$$

Now we compute the resolution refutation. The recursive definition is the following:

$$\gamma _1$$: 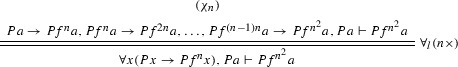
$$\gamma _n$$: 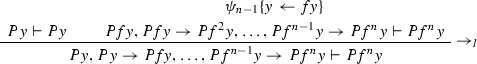 We obtain $$\Vert \gamma _{n} \Vert = \Vert \gamma _{n-1} \Vert + 3$$.

Thus, the resolution schema is $\delta _n$: 
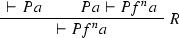
 with substitutions $\{y \leftarrow a\}, \ldots , \{y \leftarrow f^{(n-1)n}a\}$. We then get

$$\begin{aligned} \Vert \delta _{n} \Vert = 3n + 2. \end{aligned}$$

Concerning the projections we adapt an improved version, *minimal projections*, which is also used in the implementation of CERES. In this form of projection it is sufficient to derive just a subsequent of the end-sequent which reduces the number of weakenings and contractions in the ACNF.

$$\varphi _{n}[C_{1,n}]$$: 
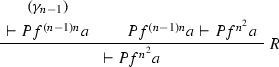
$$\varphi _{n}[C_{2}]$$ (minimal projection) 
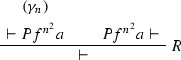
$$\varphi _{n}[C_{3}]$$ (minimal projection): 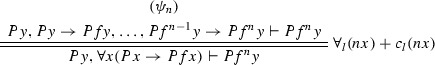 Now we can construct the ACNF. $\varphi ^{*}_n$ is the ACNF-schema:

$$\varphi ^{*}_1$$: 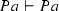
$$\varphi ^{*}_n$$: 
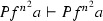
 where $F = \forall x (Px \rightarrow Pfx)$. In $\varphi ^{*}_n$ there are *n* substitution instances of the proof $\psi _{n}$ and therefore the size of the ACNF-schema $\varphi ^{*}_n$ is

$$\begin{aligned} \Vert \varphi ^{*}_n \Vert \ge a_1*n^3. \end{aligned}$$

As $C_{\mathrm{EXP}}(\varphi _n) \ge \Vert \varphi ^{*}_n \Vert$ (the algorithm $\mathrm{EXP}$ contains the construction of $\varphi ^{*}_n$) we finally obtain

$$\begin{aligned} C_{\mathrm{EXP}}(\varphi ^{*}_n) \ge a_1*n^3. \end{aligned}$$

Therefore every expansion tree extraction from $\varphi ^{*}_n$ via $\mathrm{EXP}$ is at least cubic in *n*. Now we consider the complexity of our improved method for extraction of expansion trees. We construct the projections first, here we have the complexity $O(n^{2})$ (just for $\varphi _{n}[C_{1,n}]$, otherwise constant). The construction of the refutation $\delta _n$ is in *O*(*n*). For the construction of the partial expansion proof:

$$\begin{aligned} \begin{array}{lcclc} T(y) &{} = &{} \forall x (Px \rightarrow Pfx) &{} +^y Py \rightarrow Pfy, &{}\\ &{} &{} &{} +^{fy} Pfy \rightarrow Pf^2 y, &{}\\ &{} &{} &{} \cdots &{}\\ &{} &{} &{} +^{f^{n-1}y} Pf^{n-1}y \rightarrow Pf^n y &{} \vdash \\ \end{array} \end{aligned}$$

we obtain

$$\begin{aligned} \Vert T(y) \Vert = 3(n-1) + 4 = 3n + 1. \end{aligned}$$

The last step is the computation of

$$\begin{aligned} {\mathrm{merge}}( Pa \vdash , T(y) \{y \leftarrow a\}, T(y) \{y \leftarrow f^{n}a\}, \ldots ,T(y) \{y \leftarrow f^{(n-1)n}a\}, \vdash Pf^{n^2}a) \end{aligned}$$

$O(n^2)$: concatenate sequents of complexity $3n + 1$*n*-times.

The last sequent is an expansion proof of $\varphi ^{*}_n$. The total expense of $\mathrm{EXP}_{new}$ is therefore (*k* being a constant)

$$\begin{aligned} C_{\mathrm{EXP}_{new}}(\varphi _n) \le k*(n+1)^2. \end{aligned}$$

Now we put things together and obtain that $\mathrm{EXP}_{new}$ is never more expensive than $\mathrm{EXP}$, but $\mathrm{EXP}$ cannot be linearly bounded in $\mathrm{EXP}_{new}$:

### Theorem 5

$$\mathrm{EXP}_{new}$$ outperforms $$\mathrm{EXP}$$, i.e.

- $C_{\mathrm{EXP}_{new}}(\varphi ) \le C_{\mathrm{EXP}}(\varphi )$ for all proofs $\varphi$ in $\mathbf {LK}_=$.
- There exists no constant *d* such that for all proofs $\varphi$ in $\mathbf {LK}_=$: $C_{\mathrm{EXP}}(\varphi ) \le d*C_{\mathrm{EXP}_{new}}(\varphi )$.

### Proof

(a) By Theorem 4. (b): Assume that such a constant *d* exists. By the construction of the proofs $\varphi _n$ above we would obtain

$$\begin{aligned} C_{\mathrm{EXP}}(\varphi _n)\le & {} d*C_{\mathrm{EXP}_{new}}(\varphi _n)\ \text{ for } \text{ all }\ n \ \text{ and } \text{ thus }\\ a_1*n^3\le & {} d*k*(n+1)^2 \ \text{ for } \text{ all } n. \end{aligned}$$

But $$a_1*n^3 > d*k*(n+1)^2$$ almost everywhere and we obtain a contradiction. $$\square $$

### Remark 4

We have shown that, for all proofs $\varphi$ in $\mathbf {LK}_=$ , $C_{\mathrm{EXP}_{new}}(\varphi ) \le C_{\mathrm{EXP}}(\varphi )$ and that a asymptotic speed-up of $C_{\mathrm{EXP}}$ via $C_{\mathrm{EXP}_{new}}$ is possible. The problem to define a sharp bound on $C_{\mathrm{EXP}}$ in terms of $C_{\mathrm{EXP}_{new}}$ remains open. Our conjecture is that $C_{\mathrm{EXP}}$ cannot be exponential in $C_{\mathrm{EXP}_{new}}$, i.e. that there exists a polynomial *p* such that

$$\begin{aligned} C_{\mathrm{EXP}}(\varphi ) \le p(C_{\mathrm{EXP}_{new}}(\varphi )) \text{ for } \text{ all } \varphi \text{ in } \mathbf {LK}_=. \end{aligned}$$

In addition to the gain in complexity, another gain is a compact symbolic representation of the expansion tree:

$$\begin{aligned} {\mathrm{merge}}( T_{1} \varSigma _{1}, T_{2} \varSigma _{2}, \ldots , T_{n} \varSigma _{n}). \end{aligned}$$

## Implementation and Experiments in Gapt

The algorithm $\mathrm{EXP}_{new}$ is implemented in the Gapt-system^1^ [12], which is a framework for implementing proof transformations written in the programming language Scala. Initially it was developed for the method CERES, but has been extended to other proof transformation algorithms (note that the methods described in this paper are available from version 2.5 on). This section explains how to run the described algorithm, followed by a discussion of results obtained by experiments with formal proofs.

For information on how to install and use the system Gapt we refer to the Gapt User Manual.^2^ Gapt opens in a Scala interactive shell (scala>) which can be used to run all the commands provided by the system. Under “Proof Examples” there is a set of functions that generate proofs of some end-sequent. In the following demonstration we will use the proof from Example 5, which is referred to as 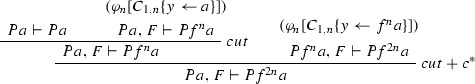 in Gapt. The sequence of commands
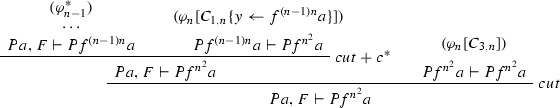
 instantiates the proof from Example 5 and stores it in the variable 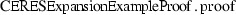, which is used as input for the method CERES. The variable 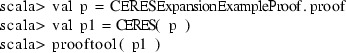 stores the generated CERES normal form. Note that the outputs stored in variables 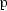 and 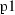 are strings representing the proofs. To obtain a proof in a tree-like structure prooftool can be used, which is a viewer for proofs and other elements also implemented in Gapt [11]. The algorithm EXP, which extracts expansion proofs from CERES normal forms is implemented in Gapt as the method 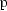. Note that this method extracts an expansion proof from any $\mathbf{LK}$-proof and not only from CERES normal forms. More information on expansion trees in Gapt can be found in [21, 25]. The following demonstration shows how to obtain expansion proofs and Herbrand sequents (corresponding to the deep function of an expansion proof) from the CERES normal form 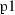 (defined in the demonstration above): 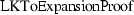 The output stored in 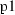 is a string representing the expansion proof of 
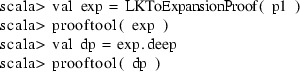
; using prooftool a better representation can be obtained. Note that also the deep function can be displayed in prooftool. The next demonstration shows how to use the algorithm $\mathrm{EXP}_{new}$, which is implemented as the method 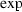 (within the CERES implementation) 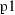 The output stored in variable 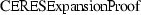 is a string representing the deep function of the expansion proof extracted from the proof projections and the corresponding ground PR refutation. Note that we use a simple built-in prover called Escargot. Instead of using Escargot, any other resolution prover supported by Gapt may be used (Gapt includes interfaces to several first-order theorem provers, such as Prover9, E Prover and LeanCoP, for more details we refer to the Gapt User Manual).

**Fig. 1:**
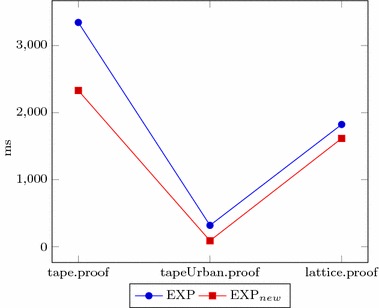
Comparison of the methods $\mathrm{EXP}$ and $\mathrm{EXP}_{new}$ for the proofs: 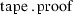, 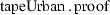, 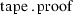

To measure the complexity of algorithms we use the command 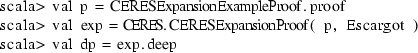, provided by the Gapt system. This command measures the time in *ms* that is needed on the system in use to perform a method. We performed several experiments with proofs containing cuts and measured the speed-up in time via 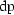 Our experiments have shown that there is a speed-up in the computation of expansion proofs with the algorithm $\mathrm{EXP}_{new}$ compared to the algorithm $\mathrm{EXP}$ already for small and simple proofs like in Example 5: our best result for the algorithm $\mathrm{EXP}$ is 47*ms*, on the other hand, with the algorithm $\mathrm{EXP}_{new}$ we obtained 18*ms*. Since even for a small and simple proof like the proof in Example 5 a speed-up is obtained, it is clear that we can increase the speed-up when we consider more complex and longer proofs. Therefore we analyzed more complicated proofs provided by Gapt, as 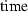, a proof of (one direction of) the equivalence of different definitions of the concept of a lattice. For background information about this proof and an informal version, we refer to Section 5 of [20]. Another interesting proof for analyzing and comparing the implemented methods is 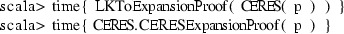, which is a proof of the statement “Given an infinite tape labelled by zeros and ones there are two cells with the same value.”. The proof proceeds by a lemma stating that on such a tape there are either infinitely many zeros or infinitely many ones. This is a subcase of the proof of the unbounded pigeonhole principle, for more information we refer to Section 4.2 of [26] and Section 3 of [1]. Note that the formalization of the tape proof as described in [26] is realized in Gapt as 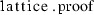. Figure 1 shows our results on experiments with the proofs 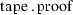, 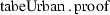 and 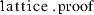; in all three cases, the method $\mathrm{EXP}_{new}$ outperforms the method $\mathrm{EXP}$.

**Table 1:** Comparison of the three different methods for the extraction of expansion proofs: based on Gentzen’s reductive method, methods $\mathrm{EXP}$ and $\mathrm{EXP}_{new}$

Proof	Reductive (ms)	$\mathrm{EXP}$ (ms)	$\mathrm{EXP}_{new}$ (ms)
fol1.proof	68	27	17
fol2.proof	53	30	21
Pi2Pigeonhole.proof	1350	680	170
Pi3Pigeonhole.proof	842	552	181
poset.proof.cycleImpliesEqual3	1621	360	147
poset.proof.cycleImpliesEqual4	6877	850	250
CutIntroduction( LinearExampleProof( 4 ))	138	70	26
CutIntroduction( LinearExampleProof( 8 ))	294	42	31
CutIntroduction( LinearExampleProof( 10 ))	577	32	28
CutIntroduction( LinearExampleProof( 15 ))	890	36	26
CutIntroduction( LinearExampleProof( 18 ))	1329	54	25
CutIntroduction( LinearExampleProof( 19 ))	1205	85	51
CutIntroduction( LinearEqExampleProof( 2 ))	190	72	32
CutIntroduction( LinearEqExampleProof( 5 ))	924	98	34
CutIntroduction( LinearEqExampleProof( 10 ))	2640	113	43
CutIntroduction( LinearEqExampleProof( 15 ))	6510	134	46
CutIntroduction( LinearEqExampleProof( 16 ))	10,875	163	53
CutIntroduction( LinearEqExampleProof( 18 ))	12,423	455	97
CutIntroduction( FactorialFunctionEqualityExampleProof( 3 ))	3525	500	360
CutIntroduction( FactorialFunctionEqualityExampleProof( 4 ))	8473	795	590
CutIntroduction( FactorialFunctionEqualityExampleProof( 5 ))	20,006	1430	930

Furthermore, we analyzed the two methods based on CERES in comparison to reductive cut-elimination methods. The Gapt system contains an implementation of Gentzen-style reductive cut-elimination, which can be used by calling the method 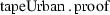. For this analysis we used several proofs provided by Gapt, as for instance simple proofs containing cuts (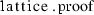, 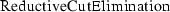), formalizations of the so-called poset proof (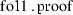) and of the pigeonhole principle (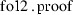, 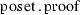) as well as some “artificial” proofs in the sense that we introduced cuts to originally cut-free proofs. Indeed, Gapt provides a cut-introduction procedure called 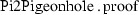, which in some cases compresses the cut-free proof 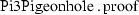 by adding cuts. We use this cut-introduction method on sequences of cut-free proofs, as for instance the sequence of proofs 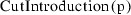, constructing cut-free proofs of sequents $P(0), \forall x (P(x) \rightarrow P(s(x))) \vdash P(s^{n}(0))$, where $n \ge 0$, 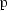, constructing proofs of $f(n) = g(n, 1)$, where *f* is the head recursive and *g* the tail recursive formulation of the factorial function and 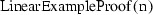, constructing cut-free proofs of sequents Refl, Trans, $\forall x (f(x) = x) \vdash f^{n}(a) = a$. Introducing cuts to these three sequences of proofs results in sequences of shorter proofs. Table 1 summarizes our results and shows that not only $\mathrm{EXP}_{new}$ is faster that $\mathrm{EXP}$, but both CERES based methods clearly outperform the method based on Gentzen-style cut-elimination.

**Fig. 2:**
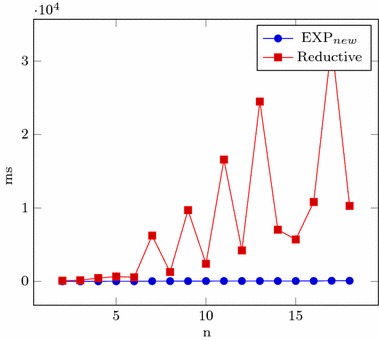
Comparison of the method $\mathrm{EXP}_{new}$ and the method Reductive for the proof 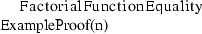 for $$2 \le n \le 18$$

**Fig. 3:**
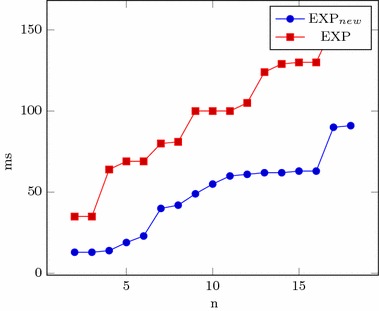
Comparison of the methods $\mathrm{EXP}$ and $\mathrm{EXP}_{new}$ for the proof (containing cuts) 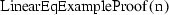 for $$2 \le n \le 18$$

Another interesting sequence of cut-free proofs provided by Gapt is formalized in 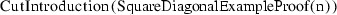, which constructs a sequence of cut-free proofs of the sequents $P(0,0), \forall x \forall y (P(x,y) \rightarrow P(s(x),y)), \forall x \forall y (P(x,y) \rightarrow P(x,s(y))) \vdash P(s^{n}(0),s^{n}(0))$, where $n \ge 0$. For every *n* the constructed proof goes along the diagonal of *P*, i.e. one x-step, then one y-step, etc. Cuts can be introduced to this sequence of proofs, however the proof containing cuts obtained by the method 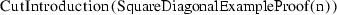 is not necessarily longer the higher the value for *n* is. More precisely, the proof 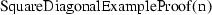 might not get as much compressed as the proof 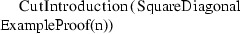 by introducing cuts, leading to a shorter proof for $n+1$. Therefore, the method based on reductive cut-elimination is not always slower for higher values of *n*. In fact, as can be observed in Fig. 2, the computing time for the method based on reductive cut-elimination on the proof 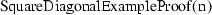 is 6235*ms*, while on the proof 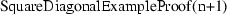 it is 1278*ms*. The analysis in Fig. 2 is performed for values $2 \le n \le 18$, as for higher values the constructed proof is too long to be analyzed on the operating system in use. Note that also for 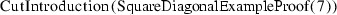 the method $\mathrm{EXP}_{new}$ outperforms the method $\mathrm{EXP}$, as can be seen in Fig. 3.

We want to remark that the obtained results depend to a great degree on the operating system in use. Therefore, it is possible that the obtained results fluctuate. Nevertheless, the speed-up of the method $\mathrm{EXP}_{new}$ compared to $\mathrm{EXP}$ is given and can be clearly recognized.

## Conclusion

In the analysis of mathematical proofs it is usually more important to gain essential mathematical information from proofs (which typically lies in the terms), than to traverse complicated and long propositional proofs. This was our motivation for avoiding the construction of an atomic cut normal form and focus on the quantifier inferences in the final proof (represented by a Herbrand sequent or by an expansion proof). In the original CERES method, the extraction of expansion proofs is performed after the final result of CERES (a proof containing at most atomic cuts, the CERES normal form) is obtained. We first analysed ACNFs and defined a new version of ACNF where the cut-inferences are the last inferences in the proof and only contractions and weakenings occur between them. Proofs of that structure are said to be in top normal form. We have shown that the logical parts of proofs in top normal form suffice to compute expansion proofs. Since proofs in CERES normal form are in top normal form, its logical parts (the proof-projections) suffice to extract expansion proofs. First we refute the characteristic clause set $\mathrm{CL}(\varphi )$ and, from a corresponding ground refutation R, obtain a set of ground substitutions $\varSigma (C)$ for all clauses $C \in \mathrm{CL}(\varphi )$ that occur in R. We construct the general CERES projections $\varphi [C]$, corresponding to the clauses $C \in \mathrm{CL}(\varphi )$ that occur in R, and extract partial expansion proofs *T*[*C*]. Finally we instantiate the partial expansion proofs *T*[*C*] by substitutions in $\varSigma (C)$ and combine the resulting expansion proofs by merging; the result is an expansion proof of the end-sequent (which coincides with the expansion proof of the CERES normal form). Using this method we avoided the construction of an atomic cut normal form.

We also obtain an improvement in asymptotic complexity. Note that we do not compute *n* instances of the projection $\varphi [C_{i}]$ with the corresponding substitution $\sigma ^{i}_{j}$ like it is needed for the construction of the CERES normal form, instead we compute the projection once, remove clause parts and then instantiate the corresponding expansion proof (Herbrand sequent) with the corresponding substitutions. The described algorithm is implemented in the Gapt system and we describe how to use it. We show that even for small and not complicated proofs a speed-up in time is obtained by the new algorithm.

Since we investigated this method for first-order logic only, further work has to deal with a generalization of this method to higher-order logic. In higher-order logic we have to deal with a different CERES-method (CERES$^{\omega }$ [19]) and a resolution calculus for higher-order logic. The extraction of expansion proofs from CERES-projections in the higher-order case is a non-trivial task, since CERES$^{\omega }$ as well as the resolution calculus for higher-order logic are much more complicated than in the first-order case. If and how the method can be generalized to the higher-order case, future work will tell.

## Footnotes

1 http://www.logic.at/gapt/.

2 http://www.logic.at/gapt/downloads/gapt-user-manual.pdf.

## References

[1] Baaz, M., Hetzl, S., Leitsch, A., Richter, C., Spohr, H.: Cut-elimination: experiments with ceres. In: International Conference on Logic for Programming Artificial Intelligence and Reasoning, pp. 481–495. Springer (2005)

[2] Baaz, M., Hetzl, S., Leitsch, A., Richter, C., Spohr, H.: Proof transformation by CERES. In: Proceedingsof 5th International Conference on Mathematical Knowledge Management, MKM 2006, Wokingham, UK, August 11–12, 2006, pp. 82–93 (2006)

[3] Baaz M, Leitsch A (1994). On skolemization and proof complexity. Fundam. Inf..

[4] Baaz M, Leitsch A (2000). Cut-elimination and redundancy-elimination by resolution. J. Symb. Comput..

[5] Baaz M, Leitsch A (2006). Towards a clausal analysis of cut-elimination. J. Symb. Comput..

[6] Baaz M, Leitsch A (2011). Methods of Cut-Elimination.

[7] Baaz M, Leitsch A (2014). Cut-elimination: syntax and semantics. Stud. Log..

[8] Berger U, Berghofer S, Letouzey P, Schwichtenberg H (2006). Program extraction from normalization proofs. Stud. Log..

[9] Berger U, Buchholz W, Schwichtenberg H (2002). Refined program extraction from classical proofs. Ann. Pure Appl. Log..

[10] Buss, S.R.: On Herbrand’s theorem. In: Leivant, D. (ed.) Logical and Computational Complexity. Selected Papers. Logic and Computational Complexity, International Workshop LCC ’94, Indianapolis, Indiana, USA, 13–16 October 1994. Lecture Notes in Computer Science, vol. 960, pp. 195–209. Springer (1994)

[11] Dunchev, C., Leitsch, A., Libal, T., Riener, M., Rukhaia, M., Weller, D., Woltzenlogel-Paleo, B.: Prooftool: a gui for the gapt framework. arXiv preprint arXiv:1307.1942 (2013)

[12] Ebner, G., Hetzl, S., Reis, G., Riener, M., Wolfsteiner, S., Zivota, S.: System description: Gapt 2.0. In: International Joint Conference on Automated Reasoning, pp. 293–301. Springer (2016)

[13] Fürstenberg H (1955). On the infinitude of the primes. Am. Math. Mon..

[14] Fürstenberg H (1978). Topological dynamics and combinatorial number theory. J. Anal. Math..

[15] Gentzen, G.: Untersuchungen über das logische Schließen. Math. Z. **39**, 176–210, 405–431 (1934–35)

[16] Girard JY (1987). Proof Theory and Logical Complexity.

[17] Gödel K (1958). Über eine bisher noch nicht benützte Erweiterung des finiten Standpunktes. Dialectica.

[18] Herbrand, J.: Recherches sur la théorie de la démonstration. PhD thesis, Université de Paris (1930)

[19] Hetzl S, Leitsch A, Weller D (2011). Ceres in higher-order logic. Ann. Pure Appl. Log..

[20] Hetzl, S., Leitsch, A., Weller, D., Paleo, B.W.: Herbrand sequent extraction. In: Autexier, S., Campbell, J.A., Rubio, J., Sorge, V., Suzuki, M., Wiedijk, F. (eds.) Proceedings of Intelligent Computer Mathematics, 9th International Conference, AISC 2008, 15th Symposium, Calculemus 2008, 7th International Conference, MKM 2008, Birmingham, UK, 28 July-1 August 2008. Lecture Notes in Computer Science, vol. 5144, pp. 462–477. Springer (2008)

[21] Hetzl, S., Libal, T., Riener, M., Rukhaia, M.: Understanding resolution proofs through Herbrands theorem. In: Galmiche, D., Larchey-Wendling, D. (eds.) Proceedings of Automated Reasoning with Analytic Tableaux and Related Methods - 22nd International Conference, TABLEAUX 2013, Nancy, France, 16–19 September 2013. Lecture Notes in Computer Science, vol. 8123, pp. 157–171. Springer (2013)

[22] Hetzl, S., Weller, D.: Expansion trees with cut. arXiv preprint arXiv:1308.0428 (2013)

[23] Miller DA (1987). A compact representation of proofs. Stud. Log..

[24] Pfenning, F.: Analytic and non-analytic proofs. In: 7th International Conference on Automated Deduction, pp. 394–413. Springer (1984)

[25] Reis, G.: Importing SMT and connection proofs as expansion trees. arXiv preprint arXiv:1507.08715 (2015)

[26] Urban, C.: Classical logic and computation. PhD thesis, University of Cambridge (2000)

[27] Van der Waerden BL (1927). Beweis einer Baudetschen Vermutung. Nieuw Arch. Wiskd..

